# Downward Mobility and Far-Right Party Support: Broad Evidence

**DOI:** 10.1177/00104140251349663

**Published:** 2025-06-27

**Authors:** Alan M. Jacobs, Mark A. Kayser

**Affiliations:** 1 8166 University of British Columbia, Vancouver, BC, Canada; 2 38962 Hertie School of Governance, Berlin, Germany

**Keywords:** far-right parties, populism, social status, class mobility

## Abstract

Much debate has centered on the relative effects of economic and cultural factors on support for far-right parties. Recent work, however, has proposed a synthesis focused on the role of *social status*, a concept capturing a combination of economic position and social esteem. While previous studies have adduced suggestive evidence that status loss shapes far-right support, this paper presents the broadest empirical assessment of the proposition to date. Focusing on long-term status change, operationalized as intergenerational occupational mobility, we find a strong relationship between mobility and support for the far right across 11 European countries. Moreover, adopting a modeling approach that addresses confounding between status levels and status change, we demonstrate an asymmetry: while downward mobility predicts increased far-right voting, upward mobility has little effect. The findings suggest that long-run economic forces that have depressed the occupational prospects of native-born workers contribute to the far right’s rise.

## Introduction

The literature on the rise of far-right parties in the advanced democratic world has been characterized by a vibrant debate about the sources of their electoral support. In broad strokes, culturalist explanations — focused on the role of citizens’ attitudes toward, variously, immigration (Ivarsflaten, 2008; Rydgren, 2008), ethno-religious minorities (Lucassen & Lubbers, 2012; Werts et al., 2013), or political elites and mainstream institutions (Ivarsflaten, 2005) — are often pitted against materialist explanations focused on the impact of economic transformations such as automation and globalization on the incomes and job security of manual workers in Western democracies (Braakmann, 2017; Han, 2016).

Recent work on the far right, however, has advanced a promising synthesis of materialist and cultural logics by focusing on the concept of *social status*. Social status can be understood as an individual’s place within a hierarchy that defines the esteem that is due to them by society. While social status is in part conditioned by one’s position within the economic order (e.g., one’s occupation and standard of living), an individual’s location within a social hierarchy is also culturally constructed and, in turn, is likely to shape their understanding of the economic and social world around them (see, e.g., Gidron & Hall, 2017). In these senses, the concept of social status may be well suited to capturing the ways in which material and cultural forces might interact to generate consequential political beliefs and attitudes.

While social status is in part a subjective and socially constructed experience, a set of economic developments over the last several decades have also reshaped the objective, material underpinnings of the social status and status changes experienced by native-born workers across OECD countries. Consider, in particular, occupational status, a key marker of social class (Breyer, 2024; Stubager et al., 2018). Importantly, the prospects for *upward* intergenerational occupational mobility have fallen dramatically in recent decades while the likelihood of *downward* intergenerational mobility has greatly increased (Breen and Müller, 2020; Bukodi & Goldthorpe, 2018; Hertel, 2017).^1^ As compared to earlier birth cohorts, children born in Western Europe and the United States since 1970 are more likely to end up in a lower-status job than their parents and less likely to end up in a higher-status one. The forces that have shaped this trend are many. Globalization, educational advancements in lower-income countries, and technological change have combined to diminish the supply of both manufacturing and higher-skilled white-collar jobs in advanced economies (Brown et al., 2010; Bukodi & Goldthorpe, 2018). Declining intergenerational occupational mobility is also a consequence of earlier gains: as manual occupational classes diminished in size and the managerial and professional salariat expanded in the immediate postwar decades, more recent cohorts have started out life in higher class strata, leaving them less room to rise and further to fall as they enter the workforce.

Several recent papers have drawn a theoretical and empirical link between social status losses among dominant groups and rising support for far-right parties. In a study of the U.S. election of 2016, for instance, Mutz (2018), argues that status anxiety among white and male voters has driven antipathy toward outgroups and susceptibility to the anti-feminist, racially tinged, xenophobic appeals of rightwing populist candidates like Donald Trump. In a similar vein, Gidron and Hall (2017) contend that a combination of economic and cultural forces has yielded a perception of status loss among white male voters in developed democracies, enhancing these voters’ receptivity to the far right’s xenophobic appeals and populist attacks on the social and political establishment. More recently, Kurer and Van Staalduinen (2022) uncover a relationship between disappointed social-status expectations and voting for the far right in the context of the 2017 German federal election.

The present paper has two goals. First, drawing on arguments in the literature, we seek to carefully theorize mechanisms through which social mobility itself — as distinct from an individual’s social-class origins or their social-class destination — might affect the likelihood of voting for far-right parties. Second, we seek to broaden and deepen our empirical understanding of the relationship between intergenerational social mobility and far-right party support. While prior studies provide suggestive evidence of a relationship between status anxiety or status loss and support for the far right, there are important limitations to the inferences that can be drawn from the current evidentiary record.

Those limitations are primarily threefold. First, existing studies have tended to focus on a single election in a single country. Given the potential idiosyncrasies of particular campaigns, parties, and candidates, a single-election focus quite dramatically limits the scope for drawing general conclusions. Second, prior studies often employ subjective status perceptions as an explanatory variable, leaving under-examined the effect of objective material drivers of social-status loss and thus making it difficult to ascertain to what degree real, structural economic changes have contributed to the far right’s rise (e.g., Gest, Reny and Mayer, 2018; Gidron & Hall, 2017; Mutz, 2018).

Third, as a matter of empirical modeling, prior studies have neglected a key substantive feature of the process of mobility itself. Mobility between status categories involves an *origin* status, a *destination* status, and a *change* between the two. Importantly, sociological theory gives reason to think that all three of these aspects of an individual’s social-status journey might matter (Sobel, 1981; Van der Waal et al., 2017). Moreover, because hierarchies have ceilings and floors, these three aspects are highly *correlated* with one another. There is thus a serious risk of confounding — between the effects of status changes and those of status levels — to which prior studies of the relationship between social status and far-right voting have rarely attended.

This paper seeks to address each of these limitations through a broad assessment of the connection between objective over-time changes in occupational class and far-right voting. First, moving beyond a single context, we examine the relationship between status loss and far-right support using individual-level European Social Survey data for 11 West European democracies between 2002 and 2020. Further, we adopt a measurement strategy devised to shed light on the effects of real economic changes that have reconfigured the social hierarchy. In particular, rather than relying on subjective reports of status loss or anxiety, we operationalize status loss (and gain) as intergenerational occupational mobility: as individuals’ movement up or down the occupational-status ladder relative to their parents’ status. This measurement choice has important consequences for what we are able to say, given our results, about the long-term *material* drivers of far-right support. Finally, we implement a modeling strategy drawn from sociological studies of mobility — the diagonal reference model — that allows a separation of the effects of upward or downward mobility from the effects of origin or destination status. Along the way, we also shed light on the mediating pathway between social status and far-right voting by estimating the effect of intergenerational occupational mobility on a set of anti-establishment and anti-immigration attitudes commonly associated with far-right appeals and support.

In sum, the paper’s analysis greatly expands the base of empirical support for the proposition that the far-right vote in advanced democracies is in part a function of changes in who sits where in the social hierarchy. Operationalizing social status in terms of objective labor-market outcomes, the paper’s results also lend broad, novel micro-level support to a particular macro-historical narrative about the rise of the far right: one in which the electoral success of the far right has in part been propelled by the long-term economic forces — including globalization, automation, and the shift to a post-industrial economy — that have limited the occupational and earning prospects of native-born workers (e.g., Broz et al., 2021; Colantone & Stanig, 2018; Gabriel et al., 2023; Iversen & Soskice, 2019; Milner, 2021).

At the same time, the findings suggest reasons for caution in drawing inferences about the links among social status change, rightwing-populist attitudes, and voting for the far right. The analysis illustrates the importance of modeling social mobility effects in a manner that is substantively grounded in the data-generating process, taking into account the possible effects of mobility’s logical correlates. Otherwise, we risk confusing the effects of early socialization or contemporary social standing for the effects of mobility itself. The findings also suggest that status loss may not operate equally on all attitudinal antecedents of far-right voting. While xenophobic opposition to immigration may be an important proximate cause of far-right support and appears to be closely associated with lower parental or own occupational status *levels*, it seems only weakly related to downward intergenerational *mobility*. In contrast, intergenerational mobility is strongly associated with the anti-system views and the *economic* concerns about immigration to which rightwing populists also regularly appeal.

## Social Status and Far-Right Support

Our focus in this paper is on *parties on the far right that combine anti-system populism with nativist electoral appeals.* While labels for such parties abound — and Mudde (2007) terms these “populist radical right” parties — we opt, for the sake of convenience, for the simpler label of *far-right* parties to describe the parties of interest in this paper.

The last decade and a half has seen a flourishing of social scientific research into the sources of far-right party support, particularly with a focus on individual-level “demand-side” factors that shape voters’ propensities to choose far-right parties and candidates at the ballot box. In this literature, cultural and economic explanations are often juxtaposed as competing accounts of the growth of far-right party support (see, e.g., Inglehart & Norris, 2016). A large swathe of studies on far-right support in OECD countries appears to find that cultural concerns and orientations – particularly, those centered around immigration, ethnic or cultural threat, and the unsettling of national identity — play a central role in shaping far-right parties’ electoral bases (e.g., Inglehart & Norris, 2016; Ivarsflaten, 2008; Lubbers & Coenders, 2017; Oesch, 2008; Werts et al., 2013).

Evidence that cultural orientations matter, however, only gets us so far in determining the importance of economic factors. Far-right party appeals are multifaceted, targeting economic concerns and cultural tensions (Ivarsflaten, 2008; Mudde, 2007), each of which may draw in different segments of the electorate. Moreover, attitudes on “cultural” issues such as immigration, the trustworthiness of national politicians and institutions, and European integration may themselves be affected by economic forces (Cochrane & Nevitte, 2014; Golder, 2016).

The search for evidence of the effect of economic adversity or insecurity on the far-right vote has turned up a mixed set of results. Perhaps the most consistent finding on this score is that the unemployed are particularly likely to vote for rightwing populists (e.g., Dehdari, 2022; Inglehart & Norris, 2016; Werts et al., 2013).^2^ Yet the literature does not yield consistent evidence that far-right support is concentrated among those at greatest socioeconomic disadvantage. Neither subjective economic insecurity (Inglehart & Norris, 2016; Werts et al., 2013) nor income loss Mutz (2018) nor low income (Rydgren, 2008; Werts et al., 2013) appear systematically related to support for the far right, though there is evidence that relative income losses are (Burgoon et al., 2018). Findings on the role of class position are conflicting, with studies finding populist attitudes or far-right voting most concentrated, variously, among manual workers (Iversen & Soskice, 2019), the petite bourgeoisie (Inglehart & Norris, 2016), or skilled manual workers (Ivarsflaten, 2005).

### Status Loss and Support for the Far Right

A number of recent studies have sought to integrate materialist and culturalist explanatory logics with a focus on the concept of *social status*. Social status is typically defined as an individual’s standing within a hierarchy that defines the esteem or honor due to them by society. According to Weber (2019/1921, p. 455), an individual’s social status can derive from a number of sources, including birth, occupation, educational attainment, and lifestyle. Social status is thus a useful concept for advancing integrative claims insofar as an individual’s social standing is conditioned both by their location within the economic order and by cultural interpretations of that hierarchy. Social status, in turn, is likely to shape both an individual’s economic prospects and the meaning that they ascribe to developments in their own material circumstances and in the economy at large.

Scholars have, in particular, focused attention on the possible electoral consequences of status *change* over time, and especially of *losses* in (or threats to) social status. We can think about status change as an individual’s movement down or up a ranked social hierarchy—for instance, from a relatively highly ranked social class to a lower-ranked social class. Conceptualizing movement within a social hierarchy necessarily also implies an origin *level* of social status and a destination *level* of social status, both of which might themselves shape political attitudes and electoral behavior. It is important, therefore, to distinguish between theoretical logics relating to the effects of origin or destination status *levels* to arguments about the effects of mobility itself.

Arguments about the effects of *social-class origins*—often understood as the social class of family of origin during childhood—are typically arguments about the political socialization processes to which an individual is exposed early in life. These processes may include both the direct transmission of values from parents to children or the more diffuse influences of the social milieu in which an individual first formed political opinions or came to understand their place in the social order (Dalton, 1982; Jennings et al., 2009). To the extent that adherence to authoritarian values is negatively associated with social-class position (as, e.g., in Lipset (1959)’s classic formulation or in Kitschelt (2012)’s logic of occupational experiences), then children born into lower social-status families may be more likely to be socialized into culturally conservative value systems and, in turn, more likely to react to social changes, such as increased immigration, by turning to far-right parties making xenophobic appeals.

Arguments about the effects of *social-class destination*—understood as an individual’s current social-class location—may operate via either a materialist or a cultural logic (De Graaf, Nieuwbeerta and Heath, 1995). Under a materialist logic, citizens form policy and political preferences in line with the economic interests of their current social stratum (e.g., of their current occupational group). In a cultural logic, individuals over time assume social identities in line with their class position and, in turn, acculturate to prevailing norms and values within that class setting.^3^ Thus, for instance, voters in working-class occupations may tend to vote for far-right parties at higher rates either because they perceive far-right policies, such as immigration restrictions, as economically beneficial to them by reducing competition for manual jobs or because, consistent with their current class milieu, they hold less liberal values and are less accepting of ethno-cultural diversity (e.g., Lucassen & Lubbers, 2012; Oesch, 2008).

By contrast, arguments about social-status mobility—the focus of the present study—zero in on the effects of status change (or risk of change) and have tended to focus on the effects of *adverse* change in status—i.e., of status loss or threat. Scholars have articulated at least four somewhat related mechanisms through which status loss might make voters more open to appeals from the far right.

First, we note instrumental arguments. As Gidron and Hall (2017) and Engler and Weisstanner (2021) contend, voters belonging to the dominant ethno-cultural group who experience or fear status decline may turn to far-right parties in the belief that those parties will act to *restore or protect their social status*. A second set of arguments connects status loss or threat to *nostalgia*. Members of a once-socially-dominant group experiencing status loss or threat may tend to view historical or longstanding social arrangements and hierarchies as more attractive and in need of defense, thereby enhancing the appeal of far-right parties’ promises to shore up or restore traditional social arrangements (see, e.g., Mutz, 2018; Gest, Reny and Mayer, 2018). Third are mechanisms that operate via heightened *xenophobia*. Drawing on large literatures in social psychology, some scholars have argued that loss (or threat of loss) of social status tends to sharpen outgroup animosity and enhance receptivity to the far right’s anti-immigrant, racially charged appeals (see, e.g., Mutz, 2018; Gidron & Hall, 2017). Fourth is a logic of *disappointment*. As Kurer and Van Staalduinen (2022) articulate this logic, when working-age adults fail to achieve a status consistent with their expectations—a standard likely to be shaped in childhood by parental status—they become disillusioned with the prospects for social advancement, blame mainstream parties and the political establishment for diminished opportunities, and become more receptive to the anti-establishment appeals of radical parties (see also Gidron & Hall, 2017).

Importantly, across all of these mobility logics, it is not where individuals start or where they end up that matters: it is the *difference* between the two. We also note that these mobility logics suggest at least the possibility that status *gain* will have the opposite effect: that those who find themselves moving up the social ladder will be *less* open either to nostalgic or xenophobic appeals or to platforms promising to upend the current order given that they experience those arrangements as working well for them.

We further note that these distinct mechanisms relate differently to different components of far-right parties’ appeals: in particular, to the nativist as compared to the anti-establishment pillars of far-right messaging. The first three logics all involve a reaction by ethnic-majority, native-born individuals against ethno-cultural diversity. The fourth mechanism, on the other hand, operates through disaffection with the prevailing political and economic order. We return to this distinction in the empirical analysis.^4^

Arguments about social-class origins, social-class destinations, and social-class mobility have distinct implications for how macro-level economic developments that have been reshaping the workforce in advanced industrialized countries might relate to the rise of the far right. As we have noted, the globalization of labor competition, technological advances in production, and postwar expansions of the managerial and professional classes have changed the occupational class structure in high-income OECD countries in ways that have substantially reduced the prospects for upward intergenerational occupational mobility and raised the likelihood of downward mobility for cohorts born since about 1970 (Breen and Müller, 2020; Brown et al., 2010; Bukodi & Goldthorpe, 2018; Hertel, 2017). Under these macro-level conditions, both social class origins and destinations might well explain variation across individuals in the likelihood of voting for the far right. Yet origin- and destination-based logics are ill-suited to helping explain the aggregate-level *rise* of the far right in recent decades, at least to the extent that they hold experience in a lower social stratum to be a driver of far-right voting. This is simply because the share of the electorate whose parents were, or who themselves are, in lower-status occupations has on the whole been *declining* for the last half century. That is to say, recent decades have seen a *shrinkage* of the very social classes that should be most supportive of the far-right under an origin- or destination-based argument. Rather, a more plausible demand-side contributor to the far right’s ascent is the widespread decline in intergenerational social *mobility* across most advanced economies. We turn now to scholarly efforts to date to assess the relationship between status loss and far-right voting at the individual level.

### Empirical Contributions to Date

Several recent papers have sought to empirically examine whether status loss or threat, among members of a once-dominant social group, shape voters’ willingness to support far-right populist parties and candidates. A prominent contribution by Mutz (2018), for instance, seeks to identify drivers of the vote for Donald Trump in the 2016 presidential election. In a multivariate regression using panel data, and including both “pocketbook” factors and measures of perceived status threat, Mutz finds that changes in the latter are strongly related to changes in support for the Republican candidate while changes in personal economic hardship bear no significant relationship to this outcome.

We note three important features of this analysis that also characterize other studies of status loss and that shape what can and cannot be learned from the findings. First is the paper’s single-country, single-election (and, in fact, single-candidate) focus, which leaves open whether the revealed pattern is a general one. Second, in assessing possible materialist drivers of the Trump vote, the study speaks to the potential effects of *short-term* changes in individuals’ economic circumstances, specifically those occurring within a single election cycle, but not to the possible effects of *long-term* economic trajectories. Third, Mutz’s operationalization of status threat is grounded in subjective measures of *perceived* status threat (see also Gest, Reny and Mayer, 2018). In the next section, we address in greater detail the difference between subjective and objective status change measures. We simply note here that Mutz’s focus on perceived status threat may shed light on the psychological processes influencing the far-right vote but limits the study’s ability to speak to the potential role of real economic developments that shape individuals’ positions within the socio-economic hierarchy.

In another influential study, Gidron and Hall (2017) examine long-term losses in social status, particularly the possibility that such losses among white men in OECD countries have created fertile ground for the far right, in part by enhancing these groups’ openness to xenophobic appeals.^5^ Drawing on International Social Survey Program (ISSP) data for a set of advanced democracies, Gidron and Hall demonstrate that subjective social status is, across individuals, negatively associated with support for the populist right, even after controlling for a range of political and economic factors. They also show that the subjective social status of low-educated men has, in the aggregate, declined over the last quarter century. While articulating a compelling theoretical account of the consequences of long-term economic and cultural developments for status anxiety, Gidron and Hall adduce no direct empirical evidence of this relationship, focusing on the more proximal link between reported status anxiety and far-right support and attitudes.

Most closely related to the present study is a paper by Kurer and Van Staalduinen (2022), who articulate a theory in which intergenerational social mobility shapes the far-right vote through a logic of disappointed expectations. To test this theory, Kurer and van Staalduinen first use German panel data to estimate a model of respondent occupational status outcomes as a function of an individual’s demographic characteristics, childhood circumstances, and father’s socio-economic characteristics. They then use occupational status predictions from the fitted model to serve as a proxy for respondents’ status *expectations*, and treat the difference between predicted and realized occupational status as a measure of status discordance. “Disappointed” status expectations, so operationalized, are associated with a higher likelihood of radical, and especially radical-right, voting.

Kurer and van Staalduinen’s analysis represents a significant step forward in the examination of the effects of social status on the support base for far-right parties, particularly in its modeling of status as a function of objective material developments, rather than merely perceived threat. The study also helpfully moves beyond a focus on short-term “pocketbook” gains and losses to assess instead the implications of long-term, intergenerational trajectories. McNeil and Haberstroh (2023), in their study of the 2016 Brexit vote, similarly tie objective measures of intergenerational class mobility to voting on an issue typically associated with far-right populism.

There are, however, important limitations to this set of analyses. First, like Mutz’s study, Kurer and Staalduinen’s and McNeil and Haberstroh’s papers draw their respective inferences entirely from voting behavior in a single election (Germany in 2017 and the UK in 2016, respectively), thus providing no leverage on broader patterns over space and time.

Second, the model specifications that these two papers employ leave inferences about status changes open to potential confounding by either origin or destination status. To see why such confounding might arise, consider, for instance, the average individual who has experienced a great deal of downward mobility and thus experienced “disappointed expectations.” We would expect that this individual will, on average, have started in a higher status category and ended up in a lower status category than the average person who has experienced no or upward mobility. Indeed, as Table 4 makes clear, drawing on the European Social Survey data that we analyze below, there is a strong negative correlation between downward mobility and current occupational status. Thus, if downwardly mobile voters are more likely to vote for the far right, then this could be for at least two reasons: because status loss itself causes more far-right voting or because *currently being* in a lower-status social position does. For similar reasons, and also reflected in Table 4, respondents whose experiences “exceeded” expectations will on average be of higher status in the current period. Confounding with status origin effects is the mirror image of this: those who experienced downward (upward) mobility will, on average, have started out in higher (lower) social positions than those who experienced no or upward mobility (see Table A4 in the SM). In short, with confounding by origin and destination not taken into account in the estimated models, the coefficient estimates on status discordance are potentially a mixture of the effects of origins, destinations, and mobility itself.

A third limitation pertains specifically to Kurer and van Staalduinen’s analysis. Kurer and van Staalduinen employ an innovative but highly indirect proxy of *ex ante* status expectations in measuring positive and negative status discordance. Drawing on predictions from a model trained on realized occupational outcomes in the 2010s, the measure retroactively imputes prior expectations to respondents’ past selves based on data that those individuals could not have observed at the time. This approach rests on the strong assumption that the relationship between parental and childhood circumstances, on the one hand, and later occupational outcomes, on the other hand, remains stable over time. Yet, major structural changes in the global and German economy since the 1960s have plausibly reconfigured that relationship in important ways.^6^ It is thus hard to know to what degree the authors’ measure is in fact capturing a gap between status expectations formed early in life and downstream outcomes.

Finally, McNeil (2025) undertakes a multi-country analysis of the effects of intergenerational mobility on far-right voting. His study uses an operationalization of mobility that is at considerable remove from the status-mobility logics articulated elsewhere in the literature. McNeil measures intergenerational social mobility as the difference in educational attainment between parents and children. Level of educational attainment is a component of many understandings of social class and, as McNeil points out, an important driver of contemporary political behavior. However, a voter assessing whether political and economic arrangements have worked for or failed them is more likely to understand their own educational attainment as an *input* into their life chances — as an investment that *they* have made in their own occupational and earnings prospects — than as an *outcome* against which to measure success or failure relative to their parents. Put differently, it is difficult to see why receiving less schooling than one’s parents did — as compared to landing in a lower-status occupation — would itself generate a sense of disappointment or disaffection from the prevailing political and economic system. Beyond this conceptual gap, empirical studies of social-class sorting find that educational attainment plays a much smaller role than do occupation or income in shaping perceptions of individuals’ places in the social hierarchy (Breyer, 2024; Stubager et al., 2018). The empirical association between educational and occupational mobility has also weakened considerably in Western European countries in recent decades (Breen and Müller, 2020; Bukodi & Goldthorpe, 2018).^7^ Thus, while McNeil demonstrates a strong and intriguing relationship between educational mobility and far-right voting, we see good reason to infer that this link arises via a mechanism different from the status-mobility logics on which we and much of the literature focus.

In summary, recent research on the role of status decline in the rise of far right support has made great strides. We have some reason to think that status loss has a substantial effect on far-right voting (Gidron & Mijs, 2019), possibly eclipsing or displacing the effect of economic variables (Mutz, 2018) and that intergenerational comparison is a key metric through which individuals gauge status loss (Kurer & Van Staalduinen, 2022). Yet, the current state of the literature also leaves open a number of important empirical questions: about the underlying economic drivers of status perceptions (Gidron & Mijs, 2019; Mutz, 2018), about how generalizable status effects are beyond a small number of individual elections (Kurer & Van Staalduinen, 2022; Mutz, 2018), and about the causal contribution of social status change itself as distinct from status start- and end-points.^8^

In the analysis that follows, we seek to advance our understanding of the effects of status change on far-right voting by examining the relationship between objective intergenerational class mobility and the vote across a broad time period and set of West European countries, employing an analytic approach that allows us to disentangle mobility effects from the effects of class origins and destinations.

## Data and Measures

We now empirically examine the link between intergenerational status change and far-right voting, drawing on data from the European Social Survey (ESS).^9^ We first describe the construction of the variables and of the sample. As a first analytical cut, we employ a linear probability model, which has the constraint that we are unable to control simultaneously for both parental occupational status and respondent’s current occupational status. To address this limitation, we then turn to estimation of diagonal reference models that allow us to distinguish between the two “level” effects (occupational status of parent and that of respondent) and “change” effects (mobility), avoiding important potential sources of confounding.

Our analyses use time-series cross-sectional data at the individual level, drawn from eleven Western European democracies (see Table 1) across 10 biennial ESS survey rounds, 2002–2020. In the analyses below, we model reported vote for, and affinity toward, far-right parties as a function of upward and downward occupational mobility and a set of controls.

**Table 1.** Far-Right Parties Included in ESS Analysis.

Country	Party
Austria	Bündnis Zukunft Österreich
	Freiheitliche Partei Österreichs
Belgium	Front National
	Vlaams Belang/Vlaams Block
Denmark	Dansk Folkeparti
	Fremskridtspartiet
Finland	Perussuomalaiset – Sannfinländarna (True Finns/Finns)
France	Front National
	Mouvement pour la France
	Rassamblement pour la France
Germany	Alternativ für Deutschland (2015 and later)
	Nationaldemokratische Partei Deutschlands
	Republikaner
Great Britain	British National Party
	U.K. Independence Party
Netherlands	List Pim Fortuyn
	Partij voor de Vrijheid (PVV - Wilders)
Norway	Fremskrittspartiet (Progress Party)
Sweden	Sverigedemokraterna
Switzerland	Schweizerische Volkspartei

### Dependent Variables and Sample

We employ two dependent variables (DVs) in the analysis. As our primary DV, we use an ESS question asking respondents about the party for which they voted in the most recent election. As a given ESS wave could be multiple years after the last election, raising questions about accuracy in recall and reporting, we supplement reported vote with responses to a question about party affinity (“which party you feel closest to”). The affinity variable offers the advantage of potentially including the full set of available parties at the time of the survey and, arguably, serves as a reasonable proxy for current vote intention.

Coding both DVs requires us to classify parties as far right. There, of course, exist several party-family classifications in the literature that include far- or radical-right categories and that arrive at somewhat differing codings. Rather than choose arbitrarily among existing classifications, we opt to aggregate across prominent classifications and to focus on parties on which there is some consensus about their far- or radical-right status. We begin with five datasets or studies that include a classification of Western European political parties as far or radical right: specifically, Armingeon et al. (2019); Bakker et al. (2015); Lubbers & Coenders (2017); Rovny & Rovny (2017); and Rooduijn & Burgoon (2018). Then, for each Western European political party coded as far or radical right in one study, we note how many of the other four studies also code that party as far or radical right, as shown in section A.3 of the Supplementary Materials (SM). We include in our sample only those parties that are (a) coded as far/radical right by at least half of the studies and (b) included as a named response choice in the ESS.^10^ We drop one party that meets these criteria, Italy’s Lega Nord, because it is also a regionalist party, implying that the party was not a viable option for voters outside of the country’s north.

Those Western European countries with at least one far-right party under the above criteria make up the potential set of countries for inclusion. This set excludes Ireland, Spain and Portugal because of the absence of far-right parties as ESS responses options in these countries during the study period. Finally, we drop Northern Ireland from the UK analysis given the distinctive party system in that region. Table 1 lists all countries included in our analysis and all parties coded as far right in our dependent variable measures of past vote and affinity. Section A.3 of the SM displays the coding exercise and the categorization of parties by each of the sources.

### Explanatory Variable: Intergenerational Occupational Mobility

We operationalize long-term change in social status as intergenerational occupational mobility: specifically, the difference between (a) an adult respondent’s (report of their) parent’s occupational status during the respondent’s childhood and (b) the respondent’s current occupational status. While there are a number of potential ways of operationalizing change in social status, the difference between parental and own occupational class very plausibly captures respondents’ lived experiences of upward or downward movement in the social hierarchy, particularly in light of the key role that occupation plays in defining placement within that hierarchy (Breyer, 2024; Stubager et al., 2018).

Before moving on, we recall that some studies of the relationship between status loss and far-right voting employ subjective, rather than objective, measures of status change (e.g., Gidron & Hall, 2017; Mutz, 2018). A subjective measure is appropriate for some analytic purposes, and our argument is fully consistent with — and, indeed, depends on — the claim that subjective perceptions of status loss and gain matter for far-right voting. Indeed, it is only through citizens’ *perceiving* a differential between their own objective status and their parents’ status that objective changes in status can, in our argument, exert their effects on voting behavior. Moreover, while subjective social status has other, non-objective determinants (Kelley & Kelley, 2009), it is also and importantly the case that objective and subjective measures of social mobility have been found to vary coherently with one another (Merllié, 2008; Miyakawa et al., 2011). Our decision to focus on an objective measure in this paper stems directly from our interest in shedding light on how real-world economic developments — in particular, those that have reshaped the occupational landscape — are affecting the far-right vote. As an explanatory variable, intergenerational occupational mobility is particularly well suited to this task. We also note that we employ an ordinal occupational scale with a small number of steps, meaning that movements between steps involve large changes that are very likely to be subjectively perceived by respondents.

The ESS asks respondents both about their own current occupation and about the occupations of their parents when the respondent was 14 years old. After mapping each occupation into a status level, we are then able to calculate an intergenerational occupational mobility score for each respondent. The ESS provides ISCO-88 occupational categories from 2002 to 2010, and ISCO-08 categories for 2012–2020 and has previously been used for research on intergenerational occupational mobility (e.g., Bukodi et al., 2020). We follow Oesch (2006), who proposes the following five-category scale for the study of social mobility, to put all occupations onto a common scale:

**Table:** 

1.	Upper and upper-middle class (large employers, self-employed and employed professionals, managers)
2.	Lower middle class (semi-professionals and associate managers)
3.	Small business owners (with or without employees)
4.	Skilled-working class (craft workers, clerks and skilled service workers)
5.	Low-skilled working class

For both respondent and parental occupation, the ESS categories provide sufficient information for mapping onto this scale, with one exception: for parental occupation, the ESS does not provide a discrete category for small business owners, so we collapse the third category of the scale into the second, resulting in a four-category scale, as more fully explained in SM section A.4.

Additionally, we face a choice about which parent’s occupation to benchmark against. We assume that the status of the household imprints on children’s expectations and thus code intergenerational occupational status change with respect to whichever parent’s occupation had higher status (using their shared status when these are the same).^11^

### Respondent-Sample Restrictions

We restrict the sample by respondents’ age, birth country, and ethnicity. The concept of intergenerational occupational mobility, for our purposes, is intended to refer to the difference between parental occupation and the respondent’s occupation in a relatively stable sense. In the years of early adulthood, current occupational status may not be well correlated with the occupational status with which an individual will “end up.” Likewise, an individual in early adulthood (say, in their early 20s) may not consider their current occupation to be indicative of their long-term socioeconomic trajectory. In particular, young-adult respondents are more likely to view low occupational status as temporary. We thus limit our sample to respondents over the age of 30, in order to focus the analysis on voters who have plausibly observed the unfolding of their occupational-status destinies. We report our main estimates with varying sample age cutoffs in Table A17 of the SM.

We further limit the sample to native-born members of the country’s majority ethnic group, both because status-loss arguments are largely intended to apply to once-dominant groups and because immigrants and members of ethnic minority groups are less likely to be drawn to the appeals of nativist parties regardless of personal economic experience. We employ listwise deletion for missing values on variables in a given model. Supplemental Table A3 shows the impact of each variable on sample size via listwise deletion, and Supplemental Tables A1 and A2 report respondent numbers for each country-round and summary statistics for the variables in our main analytic sample, respectively.

### Controls

Our strategy for reducing the risk of confounding while avoiding post-treatment bias is to control for those variables measured repeatedly in the ESS that (a) the voting literature indicates are likely to affect far-right support, (b) are also known influences on an individual’s occupational status, and (c) we can be confident are not themselves caused by our causal variable of interest, intergenerational occupational mobility. The set of variables measured in the ESS that we believe meets these three criteria is small, but includes age (which we enter as an integer value), sex (*female* = 1), and educational attainment (years). All three have been found to be strong predictors of far-right support, all are possible predictors of occupational mobility, and none is plausibly post-treatment (i.e., caused by intergenerational occupational mobility).^12^

Further, because individuals’ social-class starting point may affect both intergenerational mobility and propensity to support the far right, we include parent’s occupational status in all of our main specifications. For reasons of perfect multicollinearity, discussed further below, it is not possible to control for respondent’s current occupational status in a standard regression model that also includes parent’s status and intergenerational mobility as explanatory variables. We more fully address potential confounding by class origin and destination levels when we estimate diagonal reference models in Section 4.2.

Before moving on to our analysis, we summarize the data in relation to our main variables of interest in Table 2, showing the joint distribution of intergenerational occupational mobility and reported vote for the far right at the last election. At this simple descriptive level, we can see that the share of votes going to the far right increases with downward mobility and declines with upward mobility.

**Table 2.** The Share of Votes for the Far Right by Intergenerational Mobility in Occupational Status.

	Not Far Right	Far Right	Total
Up3	95.43	4.57	100.00
Up2	92.32	7.68	100.00
Up1	92.00	8.00	100.00
0	92.06	7.94	100.00
Down1	90.27	9.73	100.00
Down2	88.62	11.38	100.00
Down3	88.04	11.96	100.00
Total (pct)	91.55	8.45	100.00
Total (*N*)	81,692	7,539	89,231

*Note*. Respondent sample restricted to native born, majority ethnic-group members over 30 years of age in the 11 countries indicated in Table 1.

## Results

### Linear Probability Models

We begin by estimating a set of linear probability models with fixed effects for country-survey rounds.^13^ Our main dependent variable is reported vote at the last election, with a binary dependent variable indicating whether the vote was for a far-right party, with results displayed in Model 1 of Table 3. Because the vote-choice question requires respondents to recall a past action, we add a second model for robustness that replaces reported vote with a binary variable indicating that the respondent feels closest to a far-right party at the time of the survey. In each model we allow maximal flexibility in functional form by creating separate dummy variables for each level of intergenerational occupational mobility (*up1*, *up2*, *up3*, *down1*, *down2*, and *down3*), with “no change” as the reference category.

**Table 3.** The Association of Upward and Downward Intergenerational Occupational Mobility With Voting for (Model 1) and Feeling Close to (Model 2) Far-Right Parties.

	(1)		(2)	
	– Voted for –		– Close to –	
Up3	−0.088***	(0.008)	−0.088***	(0.010)
Up2	−0.045***	(0.006)	−0.047***	(0.006)
Up1	−0.020***	(0.003)	−0.023***	(0.003)
Down1	0.027***	(0.003)	0.029***	(0.004)
Down2	0.055***	(0.005)	0.059***	(0.007)
Down3	0.073***	(0.012)	0.079***	(0.015)
Parent’s occ. status	0.031***	(0.003)	0.033***	(0.004)
Age (years)	−0.001***	(0.000)	−0.001***	(0.000)
Education (years)	−0.006***	(0.000)	−0.006***	(0.001)
Female	−0.041***	(0.003)	−0.041***	(0.003)
Observations	89,231		65,210	
*R* ^2^	0.073		0.098	
Adjusted *R*^2^	0.072		0.096	

*p < .05, **p < .01, ***p < .001.

*Note*. Mobility is defined as the difference between respondent’s current occupational status and the higher of the parents’ occupational status when respondent was 14 years old. Linear probability model coefficients from models with country-essround fixed effects. ESS data, rounds 1–10 (2002–2020), for the far-right parties in 11 countries as indicated in Table 1. Sample of respondents restricted to native born, majority ethnic-group members older than 30 years of age. Reference category for up and down dummies is no intergenerational occupational status change. Standard errors clustered on (91) country-surveys in parentheses.

In Model 1, we estimate the relationship between mobility and reported vote—controlling for age, sex, educational attainment, and parent’s occupational status—for all native-born, ethnic-majority respondents over the age of 30. As we see, the pattern of parameter estimates is strongly consistent with expectations. Moving up one level on the occupational scale relative to one’s parent is associated with a 2% reduction in the probability of voting for the far right, while moving down one level is associated with a similar, nearly 3%, increase in that probability. Further, the effect sizes increase monotonically for larger occupational changes, with moving up or down two levels having larger effects than moving up or down one level, and moving up or down three levels having larger effects than moving up or down two. The rough symmetry between “up” and “down” is also notable, with the *up1* coefficient somewhat similar to the *down1* coefficient, and so on for 2 and 3 moves along the ladder (though we will see a change in this pattern later in the analysis). All relationships, moreover, are highly precisely estimated.^14^ Model 2, despite more missing observations, shows remarkably similar results when the contemporaneous question about the party to which respondents feel closest is employed.

**Figure 1.**
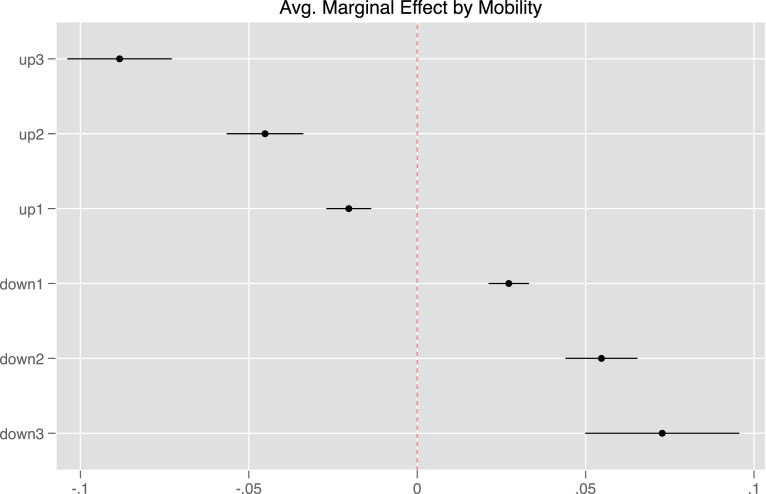
Intergenerational occupational change and far-right vote in 11 European countries. ESS data, rounds 1–10 (2002–2020). Average marginal effects in upper panel from linear probability model in Table 3. Mobility calculated relative to the higher of father’s or mother’s occupational status when respondent was 14 years old. Bars represent 95% confidence intervals. Sample restricted to respondents over 30 y.o., born in given country, and not a member of an ethnic minority.

In Figure 1, we present the evidence graphically. Based on Model 1 in Table 3, Figure 1 plots the estimated coefficients, with 95-percent confidence intervals, for each level of mobility, displaying a notably smooth pattern. Larger confidence intervals for larger shifts reflect the smaller number of respondents who experienced an extreme change, as reported in the SM.

**Figure 2.**
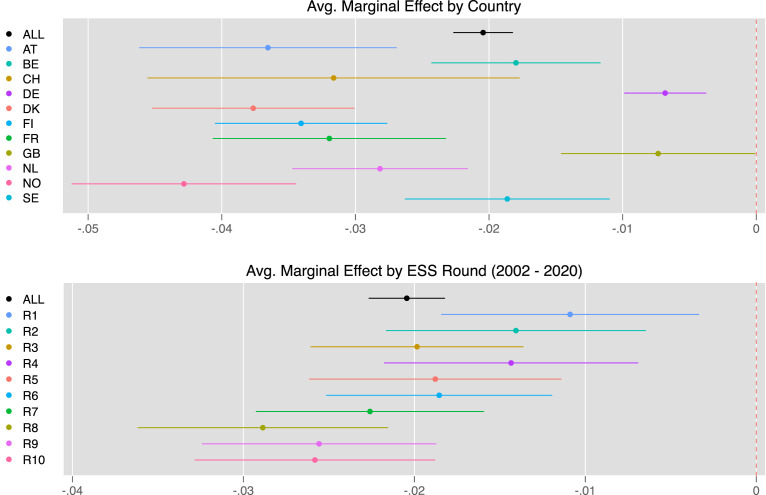
Mobility effects by country (upper panel) and ESS round (lower panel). Coefficients are based on model identical to Model 1 in Table 3 except for the replacement of the sequence of separate “up” and “down” occupational mobility dummies with a single 7-category ordinal occupational mobility variable for which positive values imply upward mobility. Mobility calculated relative to the higher of father’s or mother’s occupational status when respondent was 14 years old. Bars represent 95% confidence intervals. Sample restricted to respondents over 30 y.o., born in given country, and not a member of an ethnic minority.

In Figure 2 we examine effects in subsets of our sample. In the upper panel, we address the possibility that our results are an artefact of the estimation of a single treatment coefficient, albeit different intercepts, for all fixed-effects groups. Perhaps, for instance, countries that have more downward occupational mobility also happen to have stronger far-right parties. Based on estimates in SM Section A.7, the figure plots the results of separate, country-by-country linear probability models, where we use a single seven-point ordinal mobility variable for each country, rather than a separate dummy variable for each step up or down. The individual-level relationship between intergenerational occupational mobility and far-right voting is uniformly negative across all of the countries, albeit with notable heterogeneity. Germany and Great Britain stand out with weaker effects, possibly due to the late emergence of the AfD as a far-right populist alternative in Germany and the first-past-the-post electoral system in Britain, which has disincentivized voting for small parties such as the BNP.

To address the possibility that our results vary over time, we also examine effects within each ESS round (2002–2020). The lower panel of Figure 2 reports the LPM coefficients from regressing the individual-level vote for a far-right party on the seven-point intergenerational mobility scale, drawing on the results in Supplemental Table A8. We observe a strong, if not fully monotonic, trend toward a stronger relationship over time. Moving up a level in occupational status relative to one’s parent is uniformly negatively associated with the probability of voting for the far right but the effect is nearly 2.5 times larger in 2020 than in 2002.

### Diagonal Reference Models

While the LPM results are strong and stable, there is reason for concern about unaddressed confounding. In particular, in a model of change (e.g., mobility), it is difficult to simultaneously distinguish the effect of the starting point, the effect of the endpoint, and the effect of change itself. As discussed in section 2.2, intergenerational occupational mobility is highly correlated with both parents’ occupation and respondents’ occupation. We illustrate this correlation using our ESS sample in Table 4, which shows the correlation between the respondent’s current occupation and the intergenerational occupational mobility that they have experienced. In Table A4 in the Supplemental Material, we show parallel correlations for mobility and parents’ occupation (and also for a sample age cutoff of 35 years in Tables A5 and A6).

**Table 4.** Mobility Percentages by Respondent’s occ. status for Respondents Older Than 30 Years of Age.

	Highest	2nd	3rd	Lowest	Total
Up3	9.47	0.00	0.00	0.00	2.31
Up2	23.82	16.18	0.00	0.00	11.37
Up1	33.69	31.75	20.61	0.00	25.13
0	33.02	32.16	39.89	28.82	34.21
Down1	0.00	19.92	28.78	41.36	20.24
Down2	0.00	0.00	10.72	21.63	5.74
Down3	0.00	0.00	0.00	8.19	0.99
Total (pct)	100.00	100.00	100.00	100.00	100.00
Total (*N*)	21,723	30,725	25,970	10,813	89,231

*Note*. Intergenerational mobility (the rows) is relative to the occupation of the parent with higher status when the respondent was 14 years old. Respondent sample restricted to native born, majority ethnic-group members. Identical sample as in Model 1 in Table 3.

**Figure 3.**
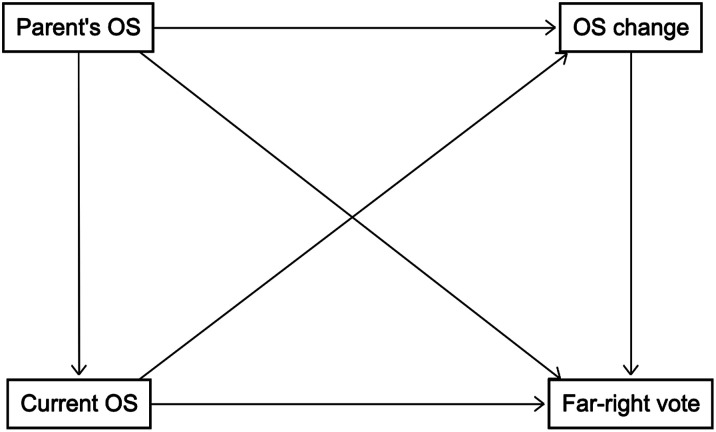
A directed acyclic graph (DAG) representing the relationship among parent’s occupational status (OS), respondent’s current OS, intergenerational change in OS, and the far-right vote.

We illustrate the problem in causal terms by representing the relationships among parent’s occupational status, respondent’s current occupational status, occupational status change, and voting for the far right as a directed acyclic graph (DAG) in Figure 3. The DAG allows for parent’s occupational status to have an effect on respondent’s current status (assuming some intergenerational transmission of class position); for parent’s and current status both to determine status change (as they must arithmetically); and for all three to have an effect on the far-right vote (allowing for the possibility of status origin, destination, and change each mattering).

Per the “backdoor criterion” (Pearl, 2009), estimating the effect of status change on the far-right vote, given this DAG, requires conditioning on both the respondent’s and their parent’s occupational status. In the linear probability models above and in the SM, we only control for parent’s status, meaning that our estimates might be confounded by the effect of the respondent’s current occupational status. However, including all three variables – parent’s status, respondent’s status, and status change – in the same regression model presents a problem: because any two of these variables fully determines the third, including all three would generate a situation of perfect multicollinearity.

We address this problem with a method developed by sociologists studying class mobility to enable the estimation of change effects while controlling for level effects of both origin and destination. As explained by Sobel (1981, 1985) and, more recently, Van der Waal et al. (2017), the *diagonal reference model* (DRM) allows for estimation of the effect of mobility, unconfounded by origin and destination, if one is willing to forego separate point estimates for origin and destination effects in favor of a combined effect for both (which is then decomposed into the share of the effect coming from each). As the distinct effects of parental and respondent status are not of direct theoretical interest for the present analysis, the DRM provides an apt solution.

The DRM employs a convex combination of the effects of the origin (*μ*_*ii*_) and the destination (*μ*_*jj*_) variables in a mobility matrix such that

(1) $$Y_{i,j,k}=\mu_{i,j}+e_{i,j,k}$$

where

(2) $$\mu_{i,j}=w\mu_{i,i}+\left(1-w\right)\mu_{j,j}\;|\;0<=w<=1.$$

*Y*_*i*,*j*,*k*_ is the value of the dependent variable in row i, column j of the mobility table, i.e., a table with each occupational category for the origin as a row and with each destination category as a column. Specifically, the subscript *i* indexes the origin category, *j* the destination category, and *kϵK* the observation index. The parameter *wϵ*[0, 1] expresses the proportion of the combined effect attributable to the origin variable (parent’s occupation) and 1 − *w* the proportion attributable to the destination variable (own occupation). With the origin and destination variables treated jointly, mobility (i.e., the difference between the two) is no longer perfectly determined and its effects can be estimated.

Equation (1) can be extended linearly to include covariates, so that

(3) $$Y_{i,j,k}=\mu_{i,j}+XB+e_{i,j,k}$$

where **X** is a covariate vector, which includes the mobility random variable, and **B** a parameter vector. For binary dependent variables, such as voting for the far right, equation (3) can be estimated via maximum likelihood with a logit link function, so that

(4) $$pr\left(Y_{i,j,k}=1\right)=\frac{1}{1+e^{-\left(\mu_{i,j}+XB+e_{i,j,k}\right)}}$$

Table 5 presents in two segments (top and bottom) the results of estimation of a DRM of reported vote for the far right at the last election, using the package developed by Kaiser (2018). The top section reports the joint effects of the two occupation *level* variables. We can see that, for each occupational category, the same estimate is reported for both parent and respondent. We have sacrificed the ability to differentiate between origin and destination effects in favor of being able to estimate mobility effects while controlling for both. The *w* parameter does, however, provide a sense of which level variable matters most, with the *w* estimate indicating that roughly one-third of the joint effect comes from the parent’s occupational status and the remainder from the respondent’s own occupational status. Moreover, we can see that the odds of voting for the far right increase as (some combination of parent’s and own) occupational status decreases (with level 4 being the lowest status).

**Table 5.** Diagonal Reference Model With Logit Link Function.

Row (origin)		
*Occ*. *status parent* 1 (*high*)	−0.824***	(0.04)
*Occ*. *status parent* 2	−0.078*	(0.03)
*Occ*. *status parent* 3	0.412***	(0.03)
*Occ*. *status parent* 4 (*low*)	0.490***	(0.04)
Col (destination)		
*Occ*. *status self* 1 (*high*)	−0.824***	(0.04)
*Occ*. *status self* 2	−0.078*	(0.03)
*Occ*. *status self* 3	0.412***	(0.03)
*Occ*. *status self* 4 (*low*)	0.490***	(0.04)
w		
*Origin*	0.360***	(0.06)
1-w		
*Destination*	0.640***	(0.06)
xb		
*Up*3	−0.183	(0.13)
*Up*2	0.056	(0.07)
*Up*1	−0.003	(0.04)
*Down*1	0.159***	(0.04)
*Down*2	0.343***	(0.08)
*Down*3	0.459**	(0.15)
*Age* (*years*)	−0.011***	(0.00)
*Education* (*years*)	−0.078***	(0.00)
*Female*	−0.564***	(0.03)
*Constant*	−0.455***	(0.09)
*Observations*	89,231	
*BIC*	46,880	

*p < .05, **p < .01, ***p < .001.

*Note*. Effect of parent’s and own occupational status levels and intergenerational occupational change on reported vote for far-right party at last election. ESS data, rounds 1–10 (2002–2010) for the far-right parties in the 11 sample countries. Sample of respondents restricted to native born, majority ethnic-group members older than 30 years of age. Country fixed effects. Standard errors in parentheses.

So what is the effect of occupational mobility, controlling for both parent’s and respondent’s occupation? Avoiding the confounding due to the omission of respondents’ own occupational status yields mobility estimates, as shown in the lower half of Table 5, that deviate notably in some aspects from those in Table 3. Most importantly, we see that effects are now asymmetric: downward mobility has a statistically significant and positive relationship with far-right voting, but upward mobility has no relationship.

**Figure 4.**
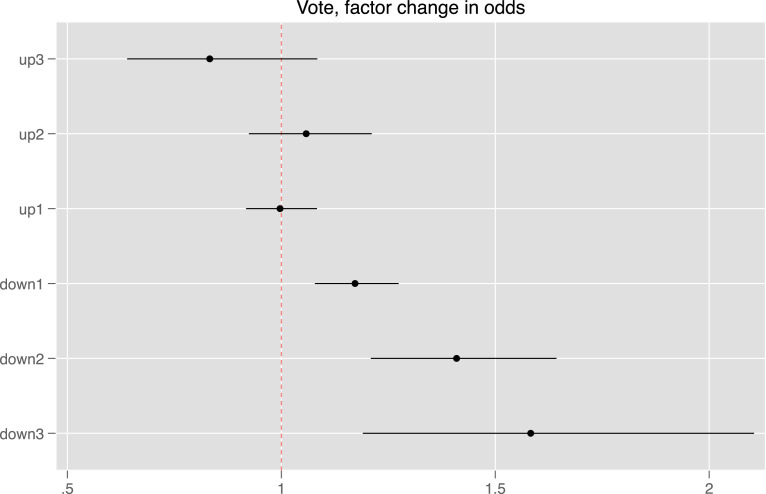
Effect intergenerational occupational change on voting for far-right party. Factor change in the odds. 95% C.I. Based on DRM model in Table 5.

Exponentiated coefficients, as shown in Figure 4, reveal that the effect of downward mobility is substantial. Dropping one occupational status category from that of one’s parent increases the odds of voting for the far right by approximately 16%, while dropping three categories corresponds to a 60% increase in the odds. Upward movements, in contrast, have no discernible effect. The different pattern of effects in the linear probability and diagonal reference models likely arises from confounding by the respondent’s occupational destination: what appears to be an effect of upward mobility in the linear probability model — and as also reported in Kurer and Van Staalduinen (2022) — is probably an effect of *ending up* in a higher-status occupation, rather than an effect of upward movement itself.

### Mechanisms

In order to shed some light on the possible mechanisms through which the mobility effect operates, we consider the effect of mobility on a set of attitudinal variables measured in the ESS that might lie between downward mobility and the decision to vote for the far right. In particular, we focus on attitudes toward immigration and on attitudes toward the political establishment, given that far-right appeals frequently involve both nativist and anti-establishment (or anti-elite) messaging. In theorizing mobility effects on the far-right vote, above, we distinguished among four theoretical logics through which these effects might operate: nostalgia, heightened xenophobia, an instrumental goal of restoring lost social status for the ethno-cultural majority, and disappointed mobility expectations. Importantly, the first three of these mechanisms operate at least in part through attitudes toward the ethno-cultural diversity brought about by immigration while the fourth primarily involves disillusionment with the political establishment and need not involve aversion to cultural diversity.

**Figure 5.**
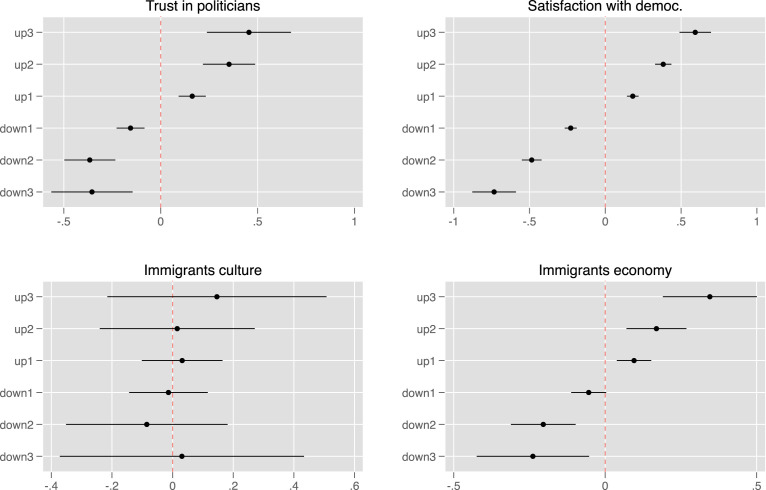
Coefficient plots of four attitude DRMs in SM Table A20. 95% CI’s.

In Figure 5, we show estimates, using diagonal reference models, of the relationship between intergenerational occupational mobility and measures of four attitudes: beliefs about whether immigrants detract from or contribute to the local culture; beliefs about whether immigrants are bad or good for the economy; trust in politicians; and satisfaction with democracy. We find markedly different results for attitudes toward immigration’s cultural effects as compared to the other three attitudes. Downward mobility is associated with lower political trust, less satisfaction with democracy, and greater concern with immigration’s *economic* effects while upward mobility is associated with attitudinal differences in the opposite direction. In contrast, *none* of the mobility variables, whether up or down, bear a relationship to cultural antipathy toward immigrants that can be statistically distinguished from zero.

We see these results as significant insofar as they help us, at least suggestively, to prise apart the different logics through which downward mobility matters: they are somewhat more consistent with the system-disaffection logic of the disappointed-expectations mechanism than they are with mechanisms in which downward mobility sharpens inter-cultural hostility or wistfulness for a more ethnically heterogeneous society.

In sum, after accounting for possible confounding by origin and destination occupational levels, we continue to find that downward intergenerational occupational mobility is associated with substantially higher rates of voting for the far right across our sample countries. The DRM results, at the same time, indicate that such effects are asymmetric: while downward mobility appears to leave voters open to far-right appeals, upward mobility does not seem to inoculate against those appeals. Moreover, after isolating mobility from origin and destination levels, we still find considerable evidence that mobility (in both directions) is associated in expected ways with anti-establishment attitudes but not with culturally focused antipathy to immigration.

### Additional Analyses

We conclude the empirical analysis by probing the robustness of our results in three respects: time period, outcome measure, and covariate inclusion. One question of interest is whether the effects of intergenerational mobility on far-right voting are consistently present over the two decades covered by our data or are concentrated in one period. We have already examined this for the LPM (lower panel of Figure 2 above and Table A14 in the SM) using year-by-year models and saw that effects in that model are strongest in the latter years. While we cannot replicate this approach with a DRM because the sample sizes become too small for the model to converge, we can split the sample into two equal-length periods, rounds 1–5 (2002–2010) and rounds 6–10 (2012–2020) as presented in Table A15 in the SM. As is readily apparent, the relationship between downward mobility and far-right voting is not detectable in the first time period but strong in the 2012–2020 period, where we observe downward-mobility effects but no upward-mobility effects.

Accounting for this over-time pattern is beyond the scope of this paper, but one potential explanation is the “mainstreaming” of the far right since the 2000s. As far-right parties’ vote share grew,^15^ these parties achieved greater representation in both legislatures and executives. They also tended to distance themselves from extremist, anti-democratic, and violent organizations on the right, granting them greater perceived credibility and social acceptance as contestants for office (Goodwin, 2019). This mainstreaming of far-right parties may well have shaped the determinants of far-right voting. While in the 2000s far-right parties would have appeared too radical or extreme even to most disillusioned voters, by the 2010s these parties were more likely to be perceived as legitimate, palatable options for citizens looking for an alternative to the political establishment.

We also examine whether our DRM results hold for our secondary outcome measure, reported affinity to (feeling close to) a far-right party. We duplicate all voting models throughout the manuscript with this alternative outcome to allay concerns that respondents might not accurately recall their vote in earlier elections. In contrast to the main DRM results in Table 5 with the “vote for” dependent variable, mobility bears no statistically discernible relationship to the affinity measure in a DRM model (with country fixed effects) estimated on all 10 survey waves. However, as shown in Table A16 in the SM, we pick up a strong relationship when focusing on the latter half of the period (rounds 6–10), with point estimates for downward mobility close to their counterparts in the “vote for” models estimated for the same time period. Upward mobility also shows modest effects in the latter time period, albeit notably weaker than the downward effects.

Finally, in the main DRM models that we have reported, we control for age, sex, educational attainment, and parent’s (origin) and respondent’s (destination) occupational status. As noted, our strategy for avoiding both confounding and post-treatment bias is to control for variables measured in the ESS that (a) are likely to affect far-right support, (b) are likely to affect occupational status, and (c) are unlikely to be caused by intergenerational occupational mobility. One further variable available in the ESS that meets these criteria is parent’s educational attainment. We do not include parent’s educational attainment in the main models because the ESS changed the scale for this measure in round 5, which would preclude the use of data from all rounds in our models. However, in Table A12 in the SM we report the results of a DRM including parent’s educational attainment for Waves 5–10. As can be seen, the asymmetric pattern persists in which downward mobility effects outstrip smaller and – for up1 and up2 – statistically insignificant effects of upward mobility.

## Conclusion

The foregoing analysis makes a number of distinctive contributions to our understanding of how social status loss shapes electoral support for far-right parties. First, the paper demonstrates, for the first time, a relationship between the intergenerational loss of occupational status and voting for the far right for a broad sample of Western European democracies across a large number of elections. The strength of this relationship appears to vary across political settings. Although the DRM does not yield sufficiently precise country-level estimates to compare effect sizes, the logit estimates suggest considerable cross-country heterogeneity. We also find that the relationship of interest has grown over time, becoming strongest after about 2010. A key takeaway from our analysis, however, is that, rather than being confined to a single country or electoral contest, the relationship between intergenerational occupational mobility and far-right voting travels widely.

Second, moving beyond subjective social status perceptions, we present micro-level evidence that long-term objective changes in economic position — intergenerational occupational mobility — bear a robust relationship to the likelihood of voting for the far right. These results suggest that the growing electoral success of the far right may in part have roots in the long-term economic forces that have constrained the occupational prospects of native-born workers, including globalization, automation, and the shift to a post-industrial economy.

Third, drawing on methods tailored to the study of social mobility, the paper demonstrates the serious risk of confounding in assessing the effect of mobility on far-right voting, with origin and destination effects potentially contaminating estimates of mobility’s effects in methods that do not distinguish between level and change effects. Finally, by taking this confounding threat into account, the paper shows that the relationship between mobility and the far-right vote — which appears symmetrical in our “naive” analysis and in prior work (e.g., Kurer and Van Staalduinen (2022)) — is in fact asymmetrical: while downward mobility is associated with increased chances of far-right voting, there is little evidence of an inoculating effect of upward mobility.

The paper’s results also have important implications for how we think about the role of short-run, as compared to long-run, economic forces in fueling the far right’s rise. We have noted a set of weak and mixed results in the literature on the effects of short-term economic loss and adversity on far-right voting. Our findings suggest that analysts seeking to understand the economy’s role in the radicalization of electorates may be unduly limiting the scope of inquiry to the extent that they focus on either static conditions (e.g., low income) or short-run fluctuations in economic welfare. If we seek the roots of fundamental shifts in political orientations, we should also be examining broad structural forces shaping the ways in which individuals’ economic destinies unfold over the long run.

Importantly, moreover, the claim that long-term economic forces matter is not an argument against the role of cultural values in shaping support for xenophobic, anti-system parties. Rather, we see the rise of the far right as likely grounded in multiple factors, some fundamentally cultural and others driven by economic forces. If, as our results suggest, downward intergenerational occupational mobility fuels anti-democratic and anti-elite sentiment, then *some* of the seemingly non-economic predicates of far-right voting may themselves have long-run economic causes. Other attitudinal drivers of far-right support — such as xenophobia — may have deeper cultural roots: indeed, we do *not* find an association between downward mobility and culturally focused anti-immigrant attitudes. Moreover, to return to a point with which we began, the reason why one’s place in the occupational landscape *matters* and defines one’s social status is itself partly cultural — a function of how societies come to allocate respect and esteem across workplace roles.

In sum, the foundations of far-right support are almost surely an admixture of “deep” culture and macro-economic forces. While some of the support base of right-wing parties is likely composed of voters acting on value orientations acquired early in life, other parts of that base, our findings imply, are composed of voters who have *learned* a set of anti-system orientations as they interact with the political economy and the social construction of status over time.

Our findings also raise important questions that merit further investigation. One such question is whether the effects of downward mobility on far-right voting varies with the nature of party appeals: if downward mobility operates via anti-establishment, rather than xenophobic attitudes, then we might expect mobility’s effects to be stronger in elections in which far-right campaigns are dominated by anti-elite or economic appeals (including those framing immigrants as an economic threat), as opposed to anti-immigrant rhetoric focused on ethno-cultural difference.

The asymmetry of mobility effects also warrants further study. This asymmetry is especially striking in juxtaposition to the symmetry of mobility’s relationship to anti-establishment attitudes, with similar point estimates for comparable upward and downward movements in those analyses. The latter results would seem to rule out explanations of the asymmetry that might operate between mobility and anti-system attitudes. It is possible that the effects of anti-system attitudes on the far-right vote are themselves asymmetrical such that pro-system beliefs fail to inoculate citizens against far-right appeals framed around other issues (e.g., immigration). We also note that we have provided only suggestive evidence that system attitudes mediate mobility’s effects: among other possibilities are that mobility affects attitudes toward the establishment but that these attitudes are not themselves a driver of the vote. Downward mobility might instead boost far-right voting, for instance, by reshaping social norms around voting (Valentim, 2024) or the salience of cultural issues (Danieli et al., 2022), rather than by changing attitudes. We leave it to future research to unpack these possibilities.

## Supplemental Material

Supplemental Material - Downward Mobility and Far-Right Party Support: Broad Evidence Supplemental Material for Downward Mobility and Far-Right Party Support: Broad Evidence by Alan M. Jacobs and Mark A. Kayser in Comparative Political Studies.

## Data Availability

Replication data and code can be found at Jacobs and Kayser (2025).

## Notes

1. When we refer to changes in intergenerational mobility over time in this paper, we mean *absolute* mobility — an individual’s prospects of moving up or down a “ladder” of social positions (e.g., occupational classes) — rather than relative mobility. While relative mobility must logically net out to zero across a population, absolute mobility can and does change over time.

2. Inglehart and Norris lump left- and rightwing populists together in their analysis. Also, in aggregate analyses, effects of the unemployment rate appear to be highly conditional on other factors. Golder (2003) finds that far-right electoral success increases as unemployment rates rise, though if and only if immigration rates are high. Jesuit et al. (2009) similarly uncover interactions among aggregate unemployment, income inequality, and immigration levels.

3. We note also the focus of some cultural arguments on the effects of educational attainment—which is closely connected to both class origins and class destinations—on far-right voting via its effects on political values (e.g., Ivarsflaten & Stubager, 2012).

4. In practice, there is some overlap between the two types of appeal, insofar as critiques of the prevailing order sometimes involve criticisms of the “open borders” purportedly favored by ruling elites. We contend, however, that the anti-establishment and xenophobic attitudes underlying far-right support have at least somewhat distinct drivers, a point that we return to empirically in the analyses in Section 4.3.

5. Gidron and Hall (2017) also propose an instrumental effect of current social status in which low-status citizens vote for far-right parties they believe to be more likely to enhance their earning prospects and status.

6. Kurer and van Staalduinen undertake a validation exercise by examining the correlation between their main status discordance measure and a more direct measure that uses verbal statements about career aspirations for a subset of younger panel respondents. While the two measures are positively and significantly correlated, an *R*^2^ of 0.263 indicates a great deal of variance in the more direct measure that is not captured by the main, indirect measure. Moreover, the authors do not report their main analyses using the more direct measure.

7. As Bukodi and Goldthorpe (2018) and the chapters in Breen and Müller (2020) show, it became more difficult for intergenerational advances in educational attainment to yield higher absolute occupational status (for children relative to their parents) following a large upward absolute shift in the occupational distribution during the postwar decades, simply because parents’ occupational starting points became, on average, much higher than they had been.

8. Two weeks prior to this manuscript’s acceptance for publication, Gugushvili et al. (2025) was published online, addressing the effects of downward occupational mobility on far-right voting. To the best of our knowledge, there existed no online version of their manuscript prior to its publication. Interested in mechanisms, Gugushvili et al. (2025) focus mostly on mediation, employing an approach critiqued by Acharya et al. (2016).

9. The online supplementary materials provide more details on sample selection. Replication data and code can be found at Jacobs and Kayser (2025).

10. This last criterion means that, for a given wave, what counts as a far-right vote will depend on which parties were present or prominent enough to warrant a named option in the ESS instrument at the time of that wave.

11. In 21% of cases in our sample, the mother had a higher occupation status, with the father having equal or higher occupatonal status in the remainder.

12. One could imagine that individuals who initially experience downward occupational mobility might consequently seek additional education to enhance their occupational prospects; however, we are observing respondent occupation, and thus intergenerational mobility, *after* the completion of that additional education, which should minimize the potential bias.

13. We avoid logit models so that we can better compare coefficients across models (Williams & Jorgensen, 2023). We repeat the analyses with only country fixed effects in SM Table A8.

14. Average marginal effects should not be confused with influence on elections, as that influence will also depend on the sizes of the relevant groups. For instance, while the *up1*, *up2*, *down1, and down2* groups are sizable, the *up3* and *down3* groups are very small (see, e.g., Table 4).

15. According to Mudde (2019), far-right parties received an average 4.7% of the vote in national elections in EU members states between 2000 and 2009, and 7.5% in the 2010 to 2018 period.
