# Supplementary material for: Downward Mobility and Far-Right Party Support: Broad Evidence
Source: Comp Polit Stud. 2025 Jun 27;59(8):1678–714. doi: 10.1177/00104140251349663 (PMC13318153; doi:10.1177/00104140251349663)
Supplement: Supplemental Material - Downward Mobility and Far-Right Party Support: Broad Evidence [file sj-pdf-1-cps-10.1177_00104140251349663.pdf]

Online Supplementary Materials

for

Alan M. Jacobs & Mark A. Kayser

"Downward Mobility and Far-Right Party Support: Broad Evidence"

April 2025

forthcoming in *Comparative Political Studies*

A Supplementary Materials

Contents

A.1 ESS Sample

The European Social Survey provides data on a few western European developed democracies that we did not include because they did not have an anti-system far-right party. Ireland does not have an anti-system far-right party of note; Italy's Lega, formerly Lega Nord, is as much a regional party as a far-right party and the Fratelli d'Italia, which is national, is only available starting in round 10. The ESS has almost no votes recorded for far-right parties in Italy. Spain had no right-wing populist parties of note until Vox emerged in the 2019 elections (captured in round 10). Not all countries participated in all rounds of the ESS, as shown in the raw frequency table below.

We originally restricted our sample to respondents who are older then 30 years of age, were born in the given country, and do not belong to a minority group (blgetmg). The ESS dropped this last variable ("Do you belong to a minority ethnic group in [country]?") from round 10 (see link, pp 9-11), replacing it with a new question (feethngr) that captured a similar concept: "Do you feel you are part of the same race or ethnic group as most people in [country]?" We consider these similar, and combine them into a single variable, so that we can include round 10 while still filtering on minority group, although the new question is not available for some countries (e.g., DE, DK, SE) in round 10, as is visible in the country-ESS round crosstab for our main analytic sample (Model 1, Table 3):

	ESS round										
Country	1	2	3	4	5	6	7	8	9	10	Total
AT	840	744	882	0	0	0	798	974	1290	871	6399
BE	718	811	881	868	788	892	896	867	792	0	7513
CH	684	736	633	528	444	500	480	531	472	458	5466
DE	1479	1266	1245	1294	1357	1413	1546	1437	1180	3763	15980
DK	930	947	987	1017	1003	1032	923	0	944	0	7783
FI	888	1029	987	1150	949	1208	1143	1036	1001	866	10257
FR	0	0	858	911	714	908	749	795	715	622	6272
GB	0	0	1078	0	0	0	993	923	1158	0	4152
NL	1355	1020	1043	983	1077	999	1060	922	855	805	10119
NO	1060	1037	996	910	900	918	819	861	805	841	9147
SE	0	0	0	0	883	1057	1017	982	941	1263	6143
Total	7954	7590	9590	7661	8115	8927	10424	9328	10153	9489	89231

Table A1: Observations in the sample for our main LPM model, Table 3, Model 1, by country and ESS round.

Variable	Obs	Mean/Prop.	Std. Dev.	Min	Max
voted for far-right party in last election	89231	.08	.28	0	1
intergenerational occ. status mobility					
up3	89231	.02	.15	0	1
up2	89231	.11	.32	0	1
up1	89231	.25	.43	0	1
updown0	89231	.34	.47	0	1
down1	89231	.2	.4	0	1
down2	89231	.06	.23	0	1
down3	89231	.01	.1	0	1
respondent occ. status					
highest	89231	.24	.43	0	1
2nd	89231	.34	.48	0	1
3rd	89231	.29	.45	0	1
lowest	89231	.12	.33	0	1
highest occ. status of parents					
highest	89231	.19	.39	0	1
2nd	89231	.3	.46	0	1
3rd	89231	.33	.47	0	1
lowest	89231	.17	.38	0	1
mother occ status higher than father	89231	.21	.41	0	1
age (years)	89231	55.46	14.34	31	102
female	89231	.5	.5	0	1
country					
AT	89231	.07	.26	0	1
BE	89231	.08	.28	0	1
CH	89231	.06	.24	0	1
DE	89231	.18	.38	0	1
DK	89231	.09	.28	0	1
FI	89231	.11	.32	0	1
FR	89231	.07	.26	0	1
GB	89231	.05	.21	0	1
NL	89231	.11	.32	0	1
NO	89231	.1	.3	0	1
SE	89231	.07	.25	0	1
ESS round					
round 1	89231	.09	.28	0	1
round 2	89231	.09	.28	0	1
round 3	89231	.11	.31	0	1
round 4	89231	.09	.28	0	1
round 5	89231	.09	.29	0	1
round 6	89231	.1	.3	0	1
round 7	89231	.12	.32	0	1
round 8	89231	.1	.31	0	1
round 9	89231	.11	.32	0	1
round 10	89231	.11	.31	0	1

Table A2: Summary statistics for the sample used in Model 1 of Table 3 in the main text.

How much does each regressor in our main LPM model (Table 2, Model 1 in the paper) contribute to sample shrinkage via listwise deletion? There are 99,824 respondents who answered the vote question and match the sample criteria on which we filter (older than 30, majority ethnic group and born in country) in the 11 countries and 10 ESS rounds of our sample. (About 30 % of respondents did not respond to the question about how they voted in the previous election). The mobility variables are the largest driver of listwise deletion, shrinking the sample by 10,022 observations. Slightly less than 70% of these mobility dropouts are caused by missing parental occupation data with the remainder presumably coming from missing respondent occupation. Age and sex have little effect on the sample size.

Variable	Obs	Missings	Feq.Missings	NonMiss	Feq.NonMiss
up3	99824	10022	10.0400	89802	89.96
up2	99824	10022	10.0400	89802	89.96
up1	99824	10022	10.0400	89802	89.96
down1	99824	10022	10.0400	89802	89.96
down2	99824	10022	10.0400	89802	89.96
down3	99824	10022	10.0400	89802	89.96
occ. status parent	99824	6815	6.8270	93009	93.17
age (yrs)	99824	324	0.3246	99500	99.68
edu (yrs)	99824	656	.6572	99168	99.34
female	99824	148	0.1483	99676	99.85

Table A3: Listwise deletion

A.2 Mobility percentages

	highest	2nd	3rd	lowest	Total
up3	0.00	0.00	0.00	13.28	2.31
up2	0.00	0.00	17.39	32.08	11.37
up1	0.00	27.09	32.78	34.54	25.13
0	42.28	36.58	34.81	20.11	34.21
down1	36.07	27.67	15.03	0.00	20.24
down2	16.42	8.66	0.00	0.00	5.74
down3	5.22	0.00	0.00	0.00	0.99
Total (pct)	100.00	100.00	100.00	100.00	100.00
Total (N)	(16962)	(27010)	(29762)	(15497)	(89231)

Table A4: (> 30 y.o.). Mobility percentages by partent's occ. status for respondents older than 30 years of age. Intergenerational mobility (the rows) is relative to the occupation of the parent with higher status when the respondent was 14 years old. Respondent sample restricted to native born, majority ethnic-group members. Identical sample as in Model 1 in Table 3.

	highest	2nd	3rd	lowest	Total
up3	0.00	0.00	0.00	13.31	2.43
up2	0.00	0.00	17.36	31.97	11.78
up1	0.00	27.06	32.67	34.52	25.44
0	42.00	36.60	34.79	20.21	33.96
down1	36.19	27.62	15.18	0.00	19.87
down2	16.50	8.72	0.00	0.00	5.55
down3	5.31	0.00	0.00	0.00	0.96
Total (pct)	100.00	100.00	100.00	100.00	100.00
Total (N)	(14767)	(24049)	(27896)	(14926)	(81638)

Table A5: (> 35 y.o.). Mobility percentages by partent's occ. status for respondents older than 35 years of age. Intergenerational mobility (the rows) is relative to the occupation of the parent with higher status when the respondent was 14 years old. Respondent sample restricted to native born, majority ethnic-group members. Identical sample as in Model 3 in Table **??** in the SM.

	highest	2nd	3rd	lowest	Total
up3	10.16	0.00	0.00	0.00	2.43
up2	24.79	17.02	0.00	0.00	11.78
up1	33.30	32.51	21.52	0.00	25.44
0	31.74	31.40	40.54	29.77	33.96
down1	0.00	19.06	27.75	41.81	19.87
down2	0.00	0.00	10.18	20.69	5.55
down3	0.00	0.00	0.00	7.74	0.96
Total (pct)	100.00	100.00	100.00	100.00	100.00
Total (N)	(19538)	(28032)	(23936)	(10132)	(81638)

Table A6: (> 35 y.o.). Mobility percentages by respondent's occ. status for respondents older than 35 years of age. Intergenerational mobility (the rows) is relative to the occupation of the parent with higher status when the respondent was 14 years old. Respondent sample restricted to native born, majority ethnic-group members. Identical sample as in Model 3 in Table **??** in the SM.

A.3 Coding parties as far-right

Party	Country	In ESS	Mean	Armingeon	CHES	L&C	R&B	R&R
BZÖ	Austria	1	0.80	1	1	0	1	1
FPÖ	Austria	1	1.00	1	1	1	1	1
FN	Belgium	1	1.00	1	1	1	1	1
VB	Belgium	1	1.00	1	1	1	1	1
FP	Denmark	1	0.50	NA	1	0	0	1
DF	Denmark	1	1.00	1	1	1	1	1
PS	Finland	1	1.00	1	1	1	1	1
RPF/MPF	France	1	0.75	NA	1	1	0	1
MNR	France	1	0.33	NA	NA	0	1	0
FN	France	1	1.00	1	1	1	1	1
AfD (pre-2015)	Germany	1	0.33	1	0	NA	0	NA
AfD (2015+)	Germany	1	1.00	1	NA	NA	NA	NA
DVU/NPD	Germany	1	1.00	1	1	1	1	1
REP	Germany	1	1.00	1	1	1	1	1
MSFT	Italy	1	0.75	NA	1	1	0	1
AN	Italy	1	0.80	1	1	0	1	1
LN	Italy	1	1.00	1	1	1	1	1
FN	Italy	0	0.33	NA	NA	1	0	0
TON	Netherlands	1	0.33	NA	NA	1	0	0
CD	Netherlands	0	0.67	1	1	0	NA	NA
LPF	Netherlands	1	0.80	1	1	0	1	1
PVV	Netherlands	1	1.00	1	1	1	1	1
FRP	Norway	1	1.00	1	NA	NA	1	1
SD	Sweden	1	1.00	1	1	NA	1	1
FPS	Switzerland	1	0.25	1	NA	0	0	0
SD	Switzerland	1	0.25	1	NA	0	0	0
SVP/UDC	Switzerland	1	1.00	1	NA	1	1	1
BNP	UK	1	1.00	NA	1	1	1	1
UKIP	UK	1	1.00	1	1	1	1	1

Table A7: Coding of far-right parties. Parties that have been classified as far right or populist right-wing radical by at least half of our five sources (a mean score of .5 or above) are considered far-right. Countries without far-right parties (e.g., Ireland and, until recently, Spain) are omitted. The sources are Armingeon et al. (2019), Bakker et al. (2015), Lubbers and Coenders (2017), Rooduijn and Burgoon (2018) and Rovny and Rovny (2017). For Armingeon et al. (2019), we use the "Right" category as a coding for far right, consistent with the codebook's definition of this category.

A.4 Coding occupational status

The ESS occupational coding for parents is at the single-digit level for waves 6 to 10:

01	Professional and technical occupations such as: doctor, teacher, engineer, artist, accountant
02	Higher administrator occupations such as: banker, executive in big business, high government official, union official
03	Clerical occupations such as: secretary, clerk, office manager, bookkeeper
04	Sales occupations such as: sales manager, shop owner, shop assistant, insurance agent
05	Service occupations such as: restaurant owner, police officer, waiter, caretaker, barber, armed forces
06	Skilled worker such as: foreman, motor mechanic, printer, tool and die maker, electrician
07	Semi-skilled worker such as: bricklayer, bus driver, cannery worker, carpenter, sheet metal worker, baker
08	Unskilled worker such as: labourer, porter, unskilled factory worker
09	Farm worker such as: farmer, farm labourer, tractor driver, fisherman

This categorization requires an adjustment of the original 5-category occupational status schema from Oesch (2006) to a four-category schema for both parents and respondents. Because the ESS parental occupational schema, unlike Oesch, does not offer a discrete category for small business owners, we combine two Oesch categories (lower middle class and small business owners), resulting in the four category schema. Specifically, we convert this 9-category ESS schema to match our modification of the Oesch (2006) schema as follows:[16]

**Original**	1	2	3	4	5	6	7	8	9
**Recoded**	1	1	2	2	2	3	3	4	4

This yields our four-category schema, for both parents and respondents:

[16] Earlier waves of the ESS employ two additional classification schemes that we also converted. See our replication files (Jacobs and Kayser, 2025).

Class	Description
1	Upper and upper-middle class (large employers, self-employed and employed professionals, managers)
2	Lower middle class and small business owners (semi-professionals, associate managers, and small business owners with or without employees)
3	Skilled-working class (craft workers, clerks and skilled service workers)
4	Low-skilled working class

A.5 LPM with country fixed-effects

	(1) – Voted for –		(2) – Close to –	
up3	-0.087***	(0.006)	-0.088***	(0.007)
up2	-0.044***	(0.004)	-0.048***	(0.004)
up1	-0.021***	(0.003)	-0.025***	(0.003)
down1	0.029***	(0.003)	0.031***	(0.003)
down2	0.057***	(0.005)	0.062***	(0.006)
down3	0.074***	(0.011)	0.082***	(0.013)
parent's occ. status	0.031***	(0.001)	0.035***	(0.002)
age (years)	-0.001***	(0.000)	-0.001***	(0.000)
education (years)	-0.005***	(0.000)	-0.006***	(0.000)
female	-0.041***	(0.002)	-0.042***	(0.002)
Observations	89231		65210	
R^2	0.056		0.064	

Table A8: *Country instead of country-essround fixed effects. Upward and downward intergenerational occupational mobility effects, LPM models. Dependent variable is voting for (Model 1) and feeling close to (Model 2) far-right parties, comparing current occupation to occupation of parent with higher status when respondent was 14 years old. ESS data for far-right parties in 11 countries as indicated in Table 1. Respondent sample restricted to native born, majority ethnic-group members older than 30 years of age. Reference category for up_ and down_ dummies is no intergenerational occupational change. Robust standard errors in parentheses.*

A.6 Alternative education measures – LPM and DRM

	(1) voted for		(2) close to	
up3	-0.122***	(0.009)	-0.126***	(0.011)
up2	-0.064***	(0.006)	-0.068***	(0.007)
up1	-0.029***	(0.004)	-0.033***	(0.004)
down1	0.037***	(0.003)	0.040***	(0.004)
down2	0.073***	(0.006)	0.079***	(0.007)
down3	0.098***	(0.012)	0.107***	(0.016)
parent's occ. status	0.045***	(0.003)	0.049***	(0.004)
age (years)	-0.001***	(0.000)	-0.001***	(0.000)
female	-0.042***	(0.004)	-0.042***	(0.004)
Observations	89585		65479	
R^2	0.068		0.091	

Table A9: *Omitting education years. Upward and downward intergenerational occupational mobility effects, LPM models with country-essround (i.e., survey) fixed effects. Dependent variable is voting for (Model 1) and feeling close to (Model 2) far-right parties, comparing current occupation to occupation of parent with higher status when respondent was 14 years old. ESS data for far-right parties in 11 countries. Respondent sample restricted to native born, majority ethnic-group members older than 30 years of age. Reference category for up_ and down_ dummies is no intergenerational occupational change. Standard errors clustered on 92 country-surveys in parentheses.* $*p < 0.05, **p < 0.01, ***p < 0.001$

	(1)		(2)	
	– voted for –		– close to –	
up3	-0.075***	(0.009)	-0.079***	(0.012)
up2	-0.041***	(0.007)	-0.048***	(0.008)
up1	-0.017***	(0.004)	-0.021***	(0.005)
down1	0.024***	(0.004)	0.027***	(0.004)
down2	0.051***	(0.006)	0.055***	(0.009)
down3	0.051**	(0.015)	0.048**	(0.017)
parent's occ. status	0.028***	(0.003)	0.031***	(0.004)
age (years)	-0.001***	(0.000)	-0.002***	(0.000)
education level	-0.005***	(0.000)	-0.006***	(0.001)
female	-0.044***	(0.004)	-0.044***	(0.004)
Observations	56451		41259	
R^2	0.071		0.099	

* $p < 0.05$, ** $p < 0.01$, *** $p < 0.001$

Table A10: *Level of education (not years) on 25-pt scale. Upward and downward intergenerational occupational mobility effects, LPM models with country-essround (i.e., survey) fixed effects. Dependent variable is voting for (Model 1) and feeling close to (Model 2) far-right parties, comparing current occupation to occupation of parent with higher status when respondent was 14 years old. ESS data for far-right parties in 11 countries. ESS rounds 1-4 drop out due to missing education level data (edulvlb). Respondent sample restricted to native born, majority ethnic-group members older than 30 years of age. Reference category for up_ and down_ dummies is no intergenerational occupational change. Standard errors clustered on 58 country-surveys in parentheses.* $*p < 0.05, **p < 0.01, ***p < 0.001$

	(1)		(2)	
	voted for		close to	
up3	-0.094***	(0.010)	-0.101***	(0.013)
up2	-0.052***	(0.008)	-0.060***	(0.009)
up1	-0.024***	(0.005)	-0.027***	(0.005)
down1	0.030***	(0.004)	0.033***	(0.005)
down2	0.059***	(0.006)	0.062***	(0.009)
down3	0.061***	(0.016)	0.059**	(0.018)
parent's occ. status	0.030***	(0.004)	0.033***	(0.005)
age (years)	-0.001***	(0.000)	-0.002***	(0.000)
education (years)	-0.006***	(0.001)	-0.007***	(0.001)
father's edu level	-0.001**	(0.000)	-0.000	(0.000)
mother's edu level	-0.001***	(0.000)	-0.001***	(0.000)
female	-0.046***	(0.005)	-0.044***	(0.005)
Observations	46302		34253	
R^2	0.072		0.100	

Table A11: *Parent's education level. Main LPM table, adding covariates for father's and mother's highest level of education attainment on 25-pt scale. ESS rounds 1 - 4 drop out due to missing education level observations. Standard errors clustered on (55) country-surveys in parentheses.* $^{*}p < 0.05$, $^{**}p < 0.01$, $^{***}p < 0.001$.

	(1)		(2)	
	– Voted for –		– Close to –	
row (origin)				
occ. status parent 1 (high)	-0.741***	(0.064)	-0.735***	(0.073)
occ. status parent 2	-0.023	(0.042)	-0.058	(0.049)
occ. status parent 3	0.426***	(0.043)	0.438***	(0.050)
occ. status parent 4 (low)	0.338***	(0.051)	0.355***	(0.059)
col (destination)				
occ. status self 1 (high)	-0.741***	(0.064)	-0.735***	(0.073)
occ. status self 2	-0.023	(0.042)	-0.058	(0.049)
occ. status self 3	0.426***	(0.043)	0.438***	(0.050)
occ. status self 4 (low)	0.338***	(0.051)	0.355***	(0.059)
w				
origin	0.572***	(0.064)	0.571***	(0.079)
1-w				
destination	0.428***	(0.064)	0.429***	(0.079)
xb				
up3	-0.327*	(0.158)	-0.363*	(0.184)
up2	-0.121	(0.074)	-0.170	(0.089)
up1	-0.077	(0.050)	-0.070	(0.059)
down1	0.222***	(0.052)	0.234***	(0.062)
down2	0.489***	(0.081)	0.480***	(0.096)
down3	0.601***	(0.181)	0.541**	(0.207)
age (years)	-0.017***	(0.001)	-0.024***	(0.002)
education level	-0.060***	(0.003)	-0.072***	(0.004)
father's edu level	-0.006	(0.004)	-0.005	(0.004)
mother's edu level	-0.010*	(0.004)	-0.014**	(0.005)
female	-0.602***	(0.035)	-0.578***	(0.041)
Constant	0.265*	(0.125)	0.834***	(0.144)
Observations	46266		34222	
BIC	24936		18439	

Table A12: DRM including respondent and parent education levels. Logit link. Effect of upward and downward intergenerational mobility in occupational status on vote for (model 1) and feeling close to (model 2) a far right party. Father's and mother's highest level of education attainment on 25-pt scale. ESS rounds 5 to 10 in 11 countries. Earlier rounds drop out due to missing education level observations. Country fixed effects. Standard errors in parentheses. Respondent sample restricted to native born, majority ethnic-group members older than 30 years of age. $^{*}p < 0.05$, $^{**}p < 0.01$, $^{***}p < 0.001$.

A.7 Country-by-country LPM models

	ALL (1)	AT (2)	BE (3)	CH (4)	DE (5)	DK (6)	FI (7)	FR v(8)	GB (9)	NL (10)	NO (11)	SE (12)
upward occ. mobility	-0.020***	-0.037***	-0.018***	-0.032***	-0.007***	-0.038***	-0.034***	-0.032***	-0.007*	-0.028***	-0.043***	-0.019***
	(0.00)	(0.00)	(0.00)	(0.01)	(0.00)	(0.00)	(0.00)	(0.00)	(0.00)	(0.00)	(0.00)	(0.00)
parent's occ. status	0.025***	0.040***	0.022***	0.082***	0.006**	0.038***	0.040***	0.039***	0.011*	0.032***	0.044***	0.015**
	(0.00)	(0.01)	(0.00)	(0.01)	(0.00)	(0.00)	(0.00)	(0.01)	(0.00)	(0.00)	(0.01)	(0.00)
age (years)	-0.001***	-0.001*	-0.001***	-0.001	-0.000***	0.001***	-0.001***	-0.001***	-0.000	-0.001***	-0.001**	-0.000
	(0.00)	(0.00)	(0.00)	(0.00)	(0.00)	(0.00)	(0.00)	(0.00)	(0.00)	(0.00)	(0.00)	(0.00)
education (years)	-0.007***	-0.004**	-0.005***	-0.014***	-0.001***	-0.005***	0.001	-0.007***	-0.004***	-0.006***	-0.010***	-0.007***
	(0.00)	(0.00)	(0.00)	(0.00)	(0.00)	(0.00)	(0.00)	(0.00)	(0.00)	(0.00)	(0.00)	(0.00)
female	-0.042***	-0.046***	-0.032***	-0.076***	-0.015***	-0.038***	-0.066***	-0.032***	-0.021**	-0.028***	-0.070***	-0.049***
	(0.00)	(0.01)	(0.01)	(0.01)	(0.00)	(0.01)	(0.01)	(0.01)	(0.01)	(0.01)	(0.01)	(0.01)
Constant	0.199***	0.125***	0.159***	0.278***	0.056***	0.047*	0.057*	0.193***	0.118***	0.181***	0.252***	0.170***
	(0.01)	(0.04)	(0.02)	(0.04)	(0.01)	(0.02)	(0.02)	(0.03)	(0.03)	(0.02)	(0.03)	(0.03)
Observations	89231	6399	7513	5466	15980	7783	10257	6272	4152	10119	9147	6143
R^2	0.028	0.021	0.024	0.058	0.007	0.038	0.029	0.032	0.012	0.031	0.054	0.027

Table A13: *Country-by-country LPM models. Dependent variable is vote for a far-right party. Occ. Mobility is a 7-point scale with higher values representing greater upward mobility. ESS data, rounds 1-10, 2002-2020, for far-right parties as indicated in Table 1. Respondent sample restricted to native born, majority ethnic-group members older than 30 years of age. Robust standard errors in parentheses.* $*p < 0.05, **p < 0.01, ***p < 0.001$.

A.8 Different time periods

		ESS Round Number									
	(All)	(1)	(2)	(3)	(4)	(5)	(6)	(7)	(8)	(9)	(10)
upward occ. mobility	-0.020***	-0.011**	-0.014***	-0.020***	-0.014***	-0.019***	-0.019***	-0.023***	-0.029***	-0.026***	-0.026***
	(0.00)	(0.00)	(0.00)	(0.00)	(0.00)	(0.00)	(0.00)	(0.00)	(0.00)	(0.00)	(0.00)
parent's occ. status	0.025***	0.009	0.016**	0.021***	0.018***	0.025***	0.025***	0.030***	0.035***	0.036***	0.033***
	(0.00)	(0.01)	(0.01)	(0.00)	(0.00)	(0.00)	(0.00)	(0.00)	(0.00)	(0.00)	(0.00)
age (years)	-0.001***	-0.000	-0.001**	-0.001***	-0.001**	-0.001***	-0.001***	-0.001***	-0.002***	-0.001***	-0.001***
	(0.00)	(0.00)	(0.00)	(0.00)	(0.00)	(0.00)	(0.00)	(0.00)	(0.00)	(0.00)	(0.00)
education (years)	-0.007***	-0.008***	-0.009***	-0.004***	-0.008***	-0.007***	-0.009***	-0.009***	-0.011***	-0.009***	-0.008***
	(0.00)	(0.00)	(0.00)	(0.00)	(0.00)	(0.00)	(0.00)	(0.00)	(0.00)	(0.00)	(0.00)
female	-0.042***	-0.034***	-0.046***	-0.038***	-0.038***	-0.034***	-0.043***	-0.034***	-0.048***	-0.052***	-0.041***
	(0.00)	(0.01)	(0.01)	(0.01)	(0.01)	(0.01)	(0.01)	(0.01)	(0.01)	(0.01)	(0.01)
Constant	0.199***	0.199***	0.220***	0.127***	0.197***	0.191***	0.222***	0.231***	0.293***	0.243***	0.213***
	(0.01)	(0.03)	(0.03)	(0.02)	(0.03)	(0.03)	(0.02)	(0.02)	(0.03)	(0.02)	(0.02)
Observations	89231	7954	7590	9590	7661	8115	8927	10424	9328	10153	9489
R^2	0.028	0.017	0.027	0.016	0.027	0.025	0.036	0.032	0.043	0.040	0.036

Table A14: *ESS round-by-round LPM models. Dependent variable is vote for a far-right party. Occ. Mobility is a 7-point scale with higher values representing greater upward mobility. ESS data, rounds 1-10, 2002-2020, for far-right parties as indicated in Table 1. Respondent sample restricted to native born, majority ethnic-group members older than 30 years of age. Robust standard errors in parentheses.* $*p < 0.05, **p < 0.01, ***p < 0.001$.

	(1) Rounds 1-5		(2) Rounds 6-10	
row (origin)				
occ. status parent 1 (high)	-0.763***	(0.07)	-0.879***	(0.05)
occ. status parent 2	-0.122**	(0.04)	-0.050	(0.04)
occ. status parent 3	0.391***	(0.04)	0.469***	(0.04)
occ. status parent 4 (low)	0.494***	(0.06)	0.460***	(0.05)
col (destination)				
occ. status parent 1 (high)	-0.763***	(0.07)	-0.879***	(0.05)
occ. status parent 2	-0.122**	(0.04)	-0.050	(0.04)
occ. status parent 3	0.391***	(0.04)	0.469***	(0.04)
occ. status parent 4	0.494***	(0.06)	0.460***	(0.05)
w				
origin	0.121	(0.13)	0.578***	(0.06)
1-w				
destination	0.879***	(0.13)	0.422***	(0.06)
xb				
up3	-0.356	(0.28)	-0.276	(0.16)
up2	0.256*	(0.13)	-0.149	(0.08)
up1	0.083	(0.08)	-0.080	(0.05)
down1	0.049	(0.08)	0.227***	(0.05)
down2	0.064	(0.16)	0.503***	(0.08)
down3	0.252	(0.26)	0.636***	(0.18)
age (years)	-0.008***	(0.00)	-0.016***	(0.00)
education (years)	-0.075***	(0.01)	-0.098***	(0.01)
female	-0.552***	(0.04)	-0.559***	(0.03)
constant	-1.314***	(0.16)	0.414***	(0.12)
Observations	40910		48321	
BIC	19464		26470	

Table A15: DRM for two time periods. Dependent variable is reported vote for a far-right party listed in Table 1. Occ. Mobility is a 7-point scale with higher values representing greater upward mobility. ESS data, rounds 1-5 (2002-2010) and rounds 6-10 (2012-2020). Respondent sample restricted to native born, majority ethnic-group members older than 30 years of age. Standard errors in parentheses. Country fixed effects. $*p < 0.05, **p < 0.01, ***p < 0.001$.

	(1) All		(2) Rounds 1-5		(3) Rounds 6-10	
row (origin)						
occ. status parent 1 (high)	-0.764***	(0.07)	-0.753***	(0.06)	-0.954***	(0.06)
occ. status parent 2	-0.251***	(0.05)	-0.267***	(0.03)	-0.074	(0.05)
occ. status parent 3	0.389***	(0.06)	0.339***	(0.06)	0.495***	(0.05)
occ. status parent 4 (low)	0.626***	(0.05)	0.681***	(0.07)	0.533***	(0.05)
col (destination)						
occ. status parent 1 (high)	-0.764***	(0.07)	-0.753***	(0.06)	-0.954***	(0.06)
occ. status parent 2	-0.251***	(0.05)	-0.267***	(0.03)	-0.074	(0.05)
occ. status parent 3	0.389***	(0.06)	0.339***	(0.06)	0.495***	(0.05)
occ. status parent 4 (low)	0.626***	(0.05)	0.681***	(0.07)	0.533***	(0.05)
w						
origin	0.017	(0.32)	-0.745	(0.79)	0.631***	(0.07)
1-w						
destination	0.983**	(0.32)	1.745*	(0.79)	0.369***	(0.07)
xb						
up3	0.277	(0.39)	1.334	(1.15)	-0.444*	(0.20)
up2	0.407	(0.30)	1.187	(0.77)	-0.210*	(0.10)
up1	0.148	(0.16)	0.539	(0.39)	-0.137*	(0.06)
down1	0.003	(0.16)	-0.373	(0.40)	0.248***	(0.06)
down2	-0.054	(0.34)	-0.938	(0.81)	0.577***	(0.10)
down3	-0.128	(0.51)	-0.971	(1.22)	0.682***	(0.20)
age (years)	-0.017***	(0.00)	-0.014***	(0.00)	-0.022***	(0.00)
education (years)	-0.088***	(0.00)	-0.074***	(0.01)	-0.110***	(0.01)
female	-0.564***	(0.03)	-0.587***	(0.05)	-0.533***	(0.04)
Constant	0.007	(0.12)	-1.254***	(0.19)	0.938***	(0.14)
Observations	65210		30044		35166	
BIC	33788		13678		19478	

Table A16: DRM for two time periods. Dependent variable is feeling close to a far-right party listed in Table 1. Occ. Mobility is a 7-point scale with higher values representing greater upward mobility. ESS data, rounds 1-5 (2002-2010) and rounds 6-10 (2012-2020). Respondent sample restricted to native born, majority ethnic-group members older than 30 years of age. Standard errors in parentheses. Country fixed effects. $*p < 0.05, **p < 0.01, ***p < 0.001$.

A.9 LPM models varying the age cut-off

	— Vote Far Right —			— Close to Far Right —		
	>25 y.o.	>30 y.o.	>35 y.o.	>25 y.o.	>30 y.o.	>35 y.o.
up3	-0.090***	-0.088***	-0.086***	-0.090***	-0.088***	-0.082***
	(0.01)	(0.01)	(0.01)	(0.01)	(0.01)	(0.01)
up2	-0.048***	-0.045***	-0.043***	-0.050***	-0.047***	-0.043***
	(0.01)	(0.01)	(0.01)	(0.01)	(0.01)	(0.01)
up1	-0.021***	-0.020***	-0.020***	-0.024***	-0.023***	-0.023***
	(0.00)	(0.00)	(0.00)	(0.00)	(0.00)	(0.00)
down1	0.027***	0.027***	0.026***	0.029***	0.029***	0.029***
	(0.00)	(0.00)	(0.00)	(0.00)	(0.00)	(0.00)
down2	0.054***	0.055***	0.052***	0.061***	0.059***	0.058***
	(0.01)	(0.01)	(0.01)	(0.01)	(0.01)	(0.01)
down3	0.073***	0.073***	0.074***	0.074***	0.079***	0.079***
	(0.01)	(0.01)	(0.01)	(0.01)	(0.02)	(0.02)
parent's occ. status	0.032***	0.031***	0.029***	0.035***	0.033***	0.030***
	(0.00)	(0.00)	(0.00)	(0.00)	(0.00)	(0.00)
age (years)	-0.001***	-0.001***	-0.001***	-0.001***	-0.001***	-0.001***
	(0.00)	(0.00)	(0.00)	(0.00)	(0.00)	(0.00)
education (years)	-0.006***	-0.006***	-0.006***	-0.007***	-0.006***	-0.006***
	(0.00)	(0.00)	(0.00)	(0.00)	(0.00)	(0.00)
female	-0.041***	-0.041***	-0.041***	-0.042***	-0.041***	-0.040***
	(0.00)	(0.00)	(0.00)	(0.00)	(0.00)	(0.00)
Constant	0.164***	0.159***	0.160***	0.210***	0.198***	0.201***
	(0.01)	(0.01)	(0.01)	(0.01)	(0.01)	(0.01)
N	95630	89231	81638	69794	65210	59909
R^2	0.074	0.073	0.072	0.102	0.098	0.095

Table A17: *By age cut-off. Upward and downward intergenerational occupational mobility effects, linear probability models with country-essround (i.e., survey) fixed effects. Both male and female respondents. Dependent variable is voting for (Models 1-3) and feeling closest to (Models 4-6) far-right parties, comparing current occupation to highest occupational status of father or mother when respondent was 14 years old. ESS data, rounds 1-10, 2002-2020, for far-right parties in 11 countries. Respondent sample restricted to native born, majority ethnic-group members older than the reported value in each column. Reference category for up_ and down_ dummies is no intergenerational occupational change. Standard errors clustered on survey in parentheses.* $^{*}p < 0.05$, $^{**}p < 0.01$, $^{***}p < 0.001$.

A.10 LPM results by sex

	—– Vote Far Right —–			—– Close to Far Right —–		
	All	Men	Women	All	Men	Women
up3	-0.088***	-0.106***	-0.065***	-0.088***	-0.114***	-0.053***
	(0.008)	(0.010)	(0.009)	(0.010)	(0.012)	(0.011)
up2	-0.045***	-0.049***	-0.043***	-0.047***	-0.053***	-0.042***
	(0.006)	(0.008)	(0.006)	(0.006)	(0.008)	(0.008)
up1	-0.020***	-0.024***	-0.017***	-0.023***	-0.031***	-0.015***
	(0.003)	(0.005)	(0.003)	(0.003)	(0.005)	(0.004)
down1	0.027***	0.030***	0.025***	0.029***	0.029***	0.029***
	(0.003)	(0.004)	(0.004)	(0.004)	(0.005)	(0.004)
down2	0.055***	0.060***	0.050***	0.059***	0.056***	0.059***
	(0.005)	(0.008)	(0.007)	(0.007)	(0.010)	(0.008)
down3	0.073***	0.071***	0.073***	0.079***	0.090***	0.068***
	(0.012)	(0.019)	(0.014)	(0.015)	(0.025)	(0.019)
parent's occ. status	0.031***	0.034***	0.028***	0.033***	0.038***	0.028***
	(0.003)	(0.003)	(0.003)	(0.004)	(0.004)	(0.004)
age (years)	-0.001***	-0.001***	-0.001***	-0.001***	-0.001***	-0.001***
	(0.000)	(0.000)	(0.000)	(0.000)	(0.000)	(0.000)
education (years)	-0.006***	-0.006***	-0.006***	-0.006***	-0.007***	-0.006***
	(0.000)	(0.001)	(0.000)	(0.001)	(0.001)	(0.001)
female	-0.041***			-0.041***		
	(0.003)			(0.003)		
Constant	0.159***	0.134***	0.136***	0.198***	0.187***	0.159***
	(0.009)	(0.012)	(0.011)	(0.014)	(0.015)	(0.017)
Observations	89231	44981	44250	65210	33823	31387
R^2	0.073	0.078	0.064	0.098	0.103	0.088
Adjusted R^2	0.072	0.076	0.062	0.096	0.101	0.085

Table A18: *Results by sex. Linear probability model coefficients from models with country-essround fixed effects. ESS data, rounds 1-10 (2002-2020), for the far-right parties in 11 countries. Sample of respondents restricted to native born, majority ethnic-group members older than 30 years of age. Reference category for up and down dummies is no intergenerational occupational status change. Standard errors clustered on (91) country-surveys in parentheses.* $^{*}p < 0.05$, $^{**}p < 0.01$, $^{***}p < 0.001$.

A.11 Intergenerational Mobility's Effects on Populist Attitudes

Here we provide details on the models and results for our analyses of the relationship between intergenerational occupational mobility and the *attitudes* that should plausibly be associated with it if mobility has an effect on voting for the far right. We focus on anti-system attitudes and anti-immigrant attitudes, both of which should be increasing with downward mobility and decreasing with upward mobility under arguments about the effect of status loss arguments on the far-right vote.

A.11.1 OLS results: attitudes

As measures of anti-establishment sentiment, we draw on ESS questions asking respondents about their level of trust in politicians and about how satisfied they are with the way democracy works in their country, allowing respondents to answer on a 0 to 10 scale (10 being the most positive). Models 1 and 2 in Table **??** show estimates for OLS models with survey (country-essround) fixed effects in which trust in politicians and satisfaction with democracy, respectively, serve as dependent variables. The sample is restricted to the same 11 countries (see Table 1), 10 ESS rounds and respondents (over 30, native born, of majority ethnicity) as in the voting analyses. Taking into account the expected reversal in sign given the coding of the DVs, the pattern of coefficients in these models is very similar to that for far-right voting, with the modest exception of an anomaly in the difference between *down2* and *down3* for trust in politicians. This similarity is driven home by the coefficient plots in panels (a) and (b) of Figure **??**. Conditional on the model's covariates, then, anti-system attitudes are more prevalent among those who have ended up worse off in occupational terms than their parents as compared to those who have ended up better off.

In Model 3, our dependent variable is an ESS question asking if a country's cultural life is undermined or enriched by the presence of immigrants, with higher values indicating more pro-immigrant sentiment. Again, the pattern repeats, with long-term downward mobility being associated with more hostile attitudes toward immigrants than long-term upward mobility. We plot these results in panel (c) of Figure **??**.

	(1) Trust pol.	(2) Sat. dem.	(3) Imm. culture	(4) Imm. econ.
up3	0.660***	0.626***	0.940***	1.152***
	(0.05)	(0.06)	(0.07)	(0.06)
up2	0.448***	0.386***	0.529***	0.662***
	(0.03)	(0.04)	(0.04)	(0.03)
up1	0.201***	0.184***	0.284***	0.326***
	(0.02)	(0.02)	(0.02)	(0.02)
down1	-0.203***	-0.233***	-0.262***	-0.293***
	(0.02)	(0.02)	(0.02)	(0.02)
down2	-0.426***	-0.476***	-0.570***	-0.628***
	(0.04)	(0.04)	(0.05)	(0.04)
down3	-0.408***	-0.707***	-0.641***	-0.741***
	(0.08)	(0.08)	(0.08)	(0.08)
parent's occ. status	-0.233***	-0.260***	-0.428***	-0.451***
	(0.02)	(0.02)	(0.02)	(0.01)
age (years)	0.009***	0.006***	-0.007***	0.004***
	(0.00)	(0.00)	(0.00)	(0.00)
education (years)	0.039***	0.038***	0.110***	0.097***
	(0.00)	(0.00)	(0.00)	(0.00)
female	0.071***	-0.139***	0.333***	-0.150***
	(0.02)	(0.02)	(0.02)	(0.02)
Constant	2.668***	5.486***	4.419***	5.226***
	(0.07)	(0.08)	(0.10)	(0.10)
Observations	88901	88442	88319	87947
R^2	0.137	0.161	0.168	0.139

Table A19: *Effect of intergenerational occupational mobility on four attitudinal variables: (1) trust in politicians, (2) how satisfied with the way democracy works in country, (3) belief about whether the country's cultural life is undermined or enriched by immigrants and (4) belief whether immigrants are bad or good for the country's economy. Dependent variables all range from 0 to 10 with 10 the most positive. ESS data, rounds 1-10, 2002-2020. Sample includes both male and female respondents over 30 years of age, native born and of majority ethnicity, for the 11 countries included in the sample. Reference category is no intergenerational occupational change. All models OLS, with standard errors clustered on survey (ess country round) in parentheses.* $*p < 0.05, **p < 0.01, ***p < 0.001$

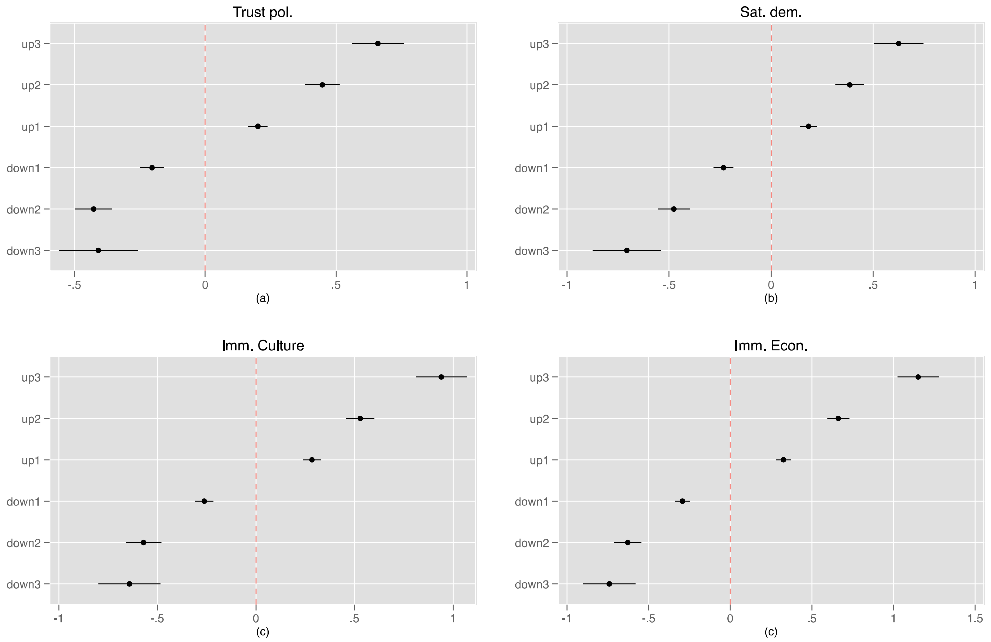

Figure A1: *Plots of coefficients representing the relationship between intergenerational occupational mobility and rightwing-populist attitudinal variables, with 95 percent confidence intervals. Estimates based on models in Table* **??**. *Control variables not plotted.*

A.11.2 DRM results: attitudes

	(1) Trust pol.		(2) Sat. dem.		(3) Imm. culture		(4) Imm. econ.	
row								
occ. status parent 1 (*high*)	0.386***	(0.02)	0.411***	(0.02)	0.638***	(0.02)	0.733***	(0.02)
occ. status parent 2	0.078***	(0.02)	0.135***	(0.01)	0.211***	(0.02)	0.174***	(0.02)
occ. status parent 3	-0.178***	(0.02)	-0.183***	(0.01)	-0.276***	(0.02)	-0.387***	(0.02)
occ. status parent 4 (*low*)	-0.286***	(0.02)	-0.363***	(0.02)	-0.573***	(0.02)	-0.520***	(0.02)
col								
occ. status self 1 (*high*)	0.386***	(0.02)	0.411***	(0.02)	0.638***	(0.02)	0.733***	(0.02)
occ. status self 2	0.078***	(0.02)	0.135***	(0.01)	0.211***	(0.02)	0.174***	(0.02)
occ. status self 3	-0.178***	(0.02)	-0.183***	(0.01)	-0.276***	(0.02)	-0.387***	(0.02)
occ. status self 4 (*low*)	-0.286***	(0.02)	-0.363***	(0.02)	-0.573***	(0.02)	-0.520***	(0.02)
w								
origin	0.847***	(0.13)	1.000	(.)	0.418**	(0.15)	0.495***	(0.05)
1-w								
destination	0.153	(0.13)	-0.000	(.)	0.582***	(0.15)	0.505***	(0.05)
xb								
*up*3	0.455***	(0.11)	0.593***	(0.05)	0.146	(0.18)	0.346***	(0.08)
*up*2	0.353***	(0.07)	0.382***	(0.03)	0.015	(0.13)	0.169***	(0.05)
*up*1	0.163***	(0.04)	0.181***	(0.02)	0.032	(0.07)	0.095***	(0.03)
*down*1	-0.156***	(0.04)	-0.228***	(0.02)	-0.014	(0.07)	-0.054	(0.03)
*down*2	-0.366***	(0.07)	-0.486***	(0.03)	-0.085	(0.14)	-0.204***	(0.05)
*down*3	-0.355***	(0.11)	-0.733***	(0.07)	0.031	(0.21)	-0.239*	(0.09)
age (*years*)	0.009***	(0.00)	0.007***	(0.00)	-0.008***	(0.00)	0.005***	(0.00)
education (*years*)	0.041***	(0.00)	0.042***	(0.00)	0.109***	(0.00)	0.102***	(0.00)
female	0.066***	(0.01)	-0.142***	(0.01)	0.330***	(0.01)	-0.154***	(0.01)
constant	2.772***	(0.05)	5.114***	(0.05)	3.827***	(0.06)	3.839***	(0.05)
sigma								
constant	2.020***	(0.00)	2.075***	(0.00)	2.169***	(0.01)	2.112***	(0.01)
observations	88901		88442		88319		87947	
BIC	377546		380396		387688		381398	

Table A20: *Diagonal reference model with linear link function. Effect of intergenerational occupational mobility on four attitudinal variables: (1) trust in politicians, (2) how satisfied with the way democracy works in country, (3) belief about whether the country's cultural life is undermined or enriched by immigrants and (4) belief about whether immigrants are bad or good for the economy. Dependent variables all range from 0 to 10 with 10 the most positive. Linear estimates from DRM with country fixed effects. ESS data, rounds 1-10, 2002-2020. Sample includes both male and female respondents over 30 years of age, native born and of majority ethnicity, for 11 countries. Reference category is no intergenerational occupational change. Origin share (w) in Model 2 constrained to 1. Standard errors in parentheses.* $*p < 0.05, **p < 0.01, ***p < 0.001$
